# Supplementary material for: Accuracy of endoscopic staging and targeted biopsies for routine gastric intestinal metaplasia and gastric atrophy evaluation study protocol of a prospective, cohort study: the estimate study
Source: BMJ Open. 2019 Sep 18;9(9):e032013. doi: 10.1136/bmjopen-2019-032013 (PMC6756576; doi:10.1136/bmjopen-2019-032013)
Supplement: Author's manuscript [file bmjopen-2019-032013.draft_revisions.pdf]

BMJ Open is committed to open peer review. As part of this commitment we make the peer review history of every article we publish publicly available.

When an article is published we post the peer reviewers' comments and the authors' responses online. We also post the versions of the paper that were used during peer review. These are the versions that the peer review comments apply to.

The versions of the paper that follow are the versions that were submitted during the peer review process. They are not the versions of record or the final published versions. They should not be cited or distributed as the published version of this manuscript.

BMJ Open is an open access journal and the full, final, typeset and author-corrected version of record of the manuscript is available on our site with no access controls, subscription charges or pay-per-view fees (http://bmjopen.bmj.com).

If you have any questions on BMJ Open's open peer review process please email info.bmjopen@bmj.com

BMJ Open

ACCURACY OF ENDOSCOPIC STAGING AND TARGETED BIOPSIES FOR ROUTINE GASTRIC INTESTINAL METAPLASIA AND GASTRIC ATROPHY EVALUATION: A PROSPECTIVE, COHORT STUDY – THE ESTIMATE STUDY -

Journal:	*BMJ Open*
Manuscript ID	bmjopen-2019-032013
Article Type:	Protocol
Date Submitted by the Author:	29-May-2019

Complete List of Authors:	Nieuwenburg, Stella; Erasmus MC University Medical Center, Gastroenterology & Hepatology<br>Waddingham, William; University College London Hospital, Endoscopy; UCL Cancer Institute<br>Graham, David; University College London Hospital, Endoscopy<br>Rodrigeuz-Justo, Manuel; UCL Cancer Institute; University College London Hospital, Pathology<br>Biermann, Katharina; Erasmus MC University Medical Center, Pathology<br>Kuipers, Ernst; Erasmus MC University Medical Center, Gastroenterology & Hepatology<br>Banks, Matthew; University College London Hospital, Endoscopy<br>Jansen, Marnix; University College London Hospital, Endoscopy; University College London Hospital, Pathology<br>Spaander, Manon; Erasmus MC University Medical Center, Gastroenterology &Hepatology

Keywords:	Endoscopy < GASTROENTEROLOGY, Gastrointestinal tumours < GASTROENTEROLOGY, PREVENTIVE MEDICINE

SCHOLARONE™
Manuscripts

ACCURACY OF ENDOSCOPIC STAGING AND TARGETED BIOPSIES FOR ROUTINE GASTRIC INTESTINAL METAPLASIA AND GASTRIC ATROPHY EVALUATION

A PROSPECTIVE, COHORT STUDY

# – THE ESTIMATE STUDY -

Stella A.V. Nieuwenburg[1]*, William W. Waddingham[2,3]*, David Graham[2], Manuel Rodrigez-Justo[3,4], Katharina Biermann[5], Ernst J. Kuipers[1], Matthew R. Banks[2], Marnix Jansen[2,4], Manon C.W. Spaander[1]

* *Authors share first authorship*

[1] *Department of Gastroenterology and Hepatology, Erasmus MC University Medical Center, Rotterdam, the Netherlands*

[2] *Department of Endoscopy, University College London Hospital, London, UK*

[3] *UCL Cancer Institute, University College London, London, UK*

[4] *Department of Pathology, University College London, London, UK*

[5] *Department of Pathology, Erasmus MC University Medical Center, Rotterdam, The Netherlands*

CORRESPONDENCE:

Manon C.W. Spaander, MD, PhD, Associate professor

Department of Gastroenterology and Hepatology (room Hs-308)

Erasmus MC University Medical Center

‘s Gravendijkwal 230, 3015 CE Rotterdam, The Netherlands

Telephone: +31 (0) 10 7035643 Fax: +31 (0) 10 7035172

E-mail: v.spaander@erasmusmc.nl

For peer review only

Abstract

Introduction Patients with chronic atrophic gastritis (CAG) and intestinal metaplasia (IM) are at risk of developing gastric adenocarcinoma. Their diagnosis and management currently relies on histopathological guidance after random endoscopic biopsy sampling (Sydney biopsy strategy). This approach has significant flaws such as under-diagnosis, poor reproducibility and poor correlation between endoscopy and histology. This prospective, international multicentre study aims to establish whether endoscopy-led risk stratification accurately and reproducibly predicts CAG and IM extent and disease stage.

Methods and analysis Patients with CAG and/or IM on standard white light endoscopy (WLE) will be prospectively identified and invited to undergo a second endoscopy performed by an expert endoscopist using enhanced endoscopic imaging techniques with virtual chromoendoscopy. Extent of CAG/IM will be endoscopically staged with enhanced imaging and compared to standard WLE. Histopathologic risk stratification through targeted biopsies will be compared to endoscopic disease staging and to random biopsy staging on WLE as a reference. At least 234 patients are required to show a 10% difference in sensitivity and accuracy between enhanced imaging endoscopy-led staging and the current biopsy-led staging protocol of gastric atrophy with a power (beta) of 80% and a 0.05 probability of a type I error (alpha).

Ethics and dissemination The study was approved by the respective Institutional Review Boards (Netherlands: MEC-2018-078; UK: 19/LO/0089). The findings will be published in peer-reviewed journals and presented at scientific meetings.

Trial registration number NTC=7661

For peer review only

Strengths and limitations of this study

- This is the first study to compare endoscopy-led risk stratification of premalignant gastric lesions using advanced imaging and targeted biopsies with white light endoscopy and random biopsies as a reference, performed at separate occasions with the endoscopist blinded to prior results
- To expand generalisability, an expert panel review has been set up to evaluate interobserver variability of endoscopic evaluation
- This study will additionally provide biobank biopsy and serum material for future biomarker analysis
- All procedures will be performed by expert endoscopists in teaching hospitals. Endoscopy-led staging should be feasible outside of expert settings to deliver actual changes in current practice. External reproducibility will be evaluated using interobserver variability of disease staging through video recordings

For peer review only

Introduction

Gastric adenocarcinoma remains a major cause of cancer mortality and is the most commonly diagnosed malignant condition of the gastrointestinal tract [1, 2, 3, 4]. Although incidence rates had previously been declining, recent studies demonstrated this differs among population subgroups. For example, an increasing incidence of gastric adenocarcinoma among young white cohorts in Western countries was objectified. This may be due to an increasing prevalence of gastric cancer precursors among younger adults, in particular chronic atrophic gastritis (CAG),intestinal metaplasia (IM), and dysplasia [5, 6]. These studies suggest that gastric cancer incidence rates may plateau or even increase again in the upcoming years. Importantly, with the exception of Japan and Korea, the majority of gastric cancers worldwide are diagnosed at a late stage. This results in a poor prognosis with a less than 30% 5-year survival [1, 2, 7]. Japan's earlier stage of diagnosis and superior 5-year survival highlight the need for earlier recognition and treatment [8].

Endoscopic recognition of the premalignant stomach has long been problematic and limited by the ability of endoscopist and the imaging tools. A previous study demonstrated that 22% of high-grade dysplastic (HGD) lesions and early gastric cancers were missed [9, 10]. A meta-analysis and systematic review of endoscopy follow-up studies confirmed that a marked proportion of early gastric cancers are missed at endoscopy [10]. Therefore, current practice uses histology based staging [11, 12]. However, endoscopic imaging has significantly improved with high-definition endoscopes and imaging enhancement technologies now routinely available. Some recent studies already suggested that accurate endoscopic staging of CAG and GIM is achievable and can robustly predict gastric adenocarcinoma risk. Importantly, the interobserver and intraobserver reproducibility characteristics of endoscopic CAG and GIM severity assessment are in experienced hands moderate to excellent [13, 14, 15, 16, 17]. These marked improvements in endoscopic technology and the shift towards an endoscopy-led approach will empower the endoscopist to risk stratify individuals with greater accuracy and decrease the already huge burden

For peer review only - http://bmjopen.bmj.com/site/about/guidelines.xhtml

placed on our endoscopy and histopathology departments. Therefore, the aim of this study is to evaluate if enhanced endoscopic imaging, including high-definition white light endoscopy and virtual chromoendoscopy, alongside targeted biopsies, provides an accurate and reproducible assessment of CAG and IM disease extent and staging, when compared to the current practice of white light endoscopy and random biopsies through the Sydney protocol biopsy strategy.

For peer review only

Methods and analysis

AIMS

The primary aim of this study is to assess the diagnostic accuracy for the endoscopic diagnosis of intestinal metaplasia in Sydney biopsy locations [18]. Study sites are located in the Netherlands and the United Kingdom. Secondary objectives are to evaluate a) reproducibility of endoscopic staging after expert review, b) reproducibility of histopathology for detection of intestinal metaplasia, c) the number of dysplastic or neoplastic lesions detected, and d) effects of inspection time of gastric mucosa on diagnostic accuracy.

DESIGN

This is a prospective, multicentre registry study on the accuracy and reproducibility of enhanced endoscopic imaging, including high-definition white light endoscopy and virtual chromoendoscopy, for the staging of chronic atrophic gastritis and intestinal metaplasia. Patients will be evaluated on separate occasions (6-12 months in between) using standard white-light endoscopy plus random biopsies (current diagnostic strategy) and then using enhanced endoscopic imaging with targeted biopsies (proposed diagnostic strategy). We will compare both approaches using histopathology as a reference and assess the accuracy and reproducibility of enhanced endoscopic imaging (**Fig 1**). The study protocol was approved by respective Institutional Review Boards (Netherlands: MEC-2018-078; UK: 19/LO/0089). The protocol has been registered in the Dutch trial register (NTR=7661).

PATIENT AND PUBLIC INVOLVEMENT

We maintained close links with patient alliances and interest groups, both in the Netherlands as well as in the UK. This close relationship informs our practice and is the basis for the current study design. We will engage closely with patient interest groups to communicate research findings and ensure that our deliverables are fit for purpose.

PARTICIPANTS

Sample size

For peer review only

For estimation of sample size we assume that the diagnosis of CAG or IM on enhanced imaging and targeted biopsies must be set with at least a 90% sensitivity with white light endoscopy and random biopsies as a reference. A power (beta) of 80% and a probability of type I error (alpha) of 0.05 will be handled. That purpose requires at least 234 patients to be recruited to show a 10% difference in sensitivity between enhanced endoscopy-led staging and the standard WLE.

Recruitment

All patients (> 18 years of age) referred to the endoscopy department for routine diagnostic upper gastrointestinal endoscopy and diagnosed with chronic atrophic gastritis or intestinal metaplasia between November 2018 and June 2020 are eligible for inclusion if able to give informed consent. Patients are excluded when having 1) gastric neoplasia not amenable to endoscopic resection, 2) no indication for Sydney biopsy staging on standard WLE, 3) significant comorbidity, 4) a coagulation disorder, 5) previous gastric surgery, or 6) are unable to complete the biopsy protocol in either endoscopy session.

INTERVENTIONS

Baseline characteristics

All patients are asked to complete a questionnaire on lifestyle factors, medical history, medication use and family history of gastric cancer.

White light endoscopy

Patients referred to the endoscopy department for upper gastrointestinal endoscopy for investigation of symptoms or for surveillance of a known condition will undergo their procedure on a standard diagnostic gastroscopy list. Patients found to have chronic atrophic gastritis or intestinal metaplasia will be prospectively identified. During the initial procedure patients will receive the current recommended practice. Current practice is to initially identify if gastric atrophy is present and to inspect the gastric mucosa for areas suspicious for dysplasia or malignancy. Any mucosal abnormalities suspicious for dysplasia or malignancy

For peer review only

are biopsied with tissue biopsies placed in separate containers. Following this, ten random biopsies are taken according to the Sydney protocol (see also **Fig. 2**): 4 quadrant biopsies of the antrum, 2 biopsies from the incisura, and 4 biopsies from the body of the stomach, respectively two from the lesser curve, and two from the greater curve.

Enhanced imaging endoscopy

Patients who opt to be recruited to the study will be invited for a second endoscopy at 6 to 12-months interval. This will be performed by one of the experts on this protocol. The endoscopists will be blinded to any previous endoscopy or biopsy results. This second endoscopy will be recorded and performed using enhanced endoscopic imaging. Given that these patients will have recently undergone a complete upper gastrointestinal endoscopy, whilst all anatomical landmarks will be viewed, the focus of this examination will be on the gastric mucosa. The endoscopist will record 1) the extent of gastric atrophy, 2) the presence and extent of intestinal metaplasia in each of the aforementioned areas. This will be done using our simplified endoscopic metaplasia scoring system (GRAHAM score) (**Table 1**). Biopsies will then be taken in the following manner: 1) Areas of intestinal metaplasia found in any of the Sydney protocol areas, 2) Sydney areas negative for gastric intestinal metaplasia will be randomly biopsied, as control, to complete the assessment, 3) Lesions suspicious for dysplasia or malignancy.

	Focal / Minimal metaplasia (<1/3 of surface coverage)	Moderate / extensive metaplasia (>1/3 surface coverage)
**Antrum & incisura**	1	2
**Lesser curve**	1	2
**Greater curve**	1	2

Table 1 Simplified endoscopic gastric intestinal metaplasia staging system: "GRAHAM Score"

For peer review only

Histopathological assessment

Each biopsy will be reviewed at the teaching hospital by one of the expert gastrointestinal histopathologists named on this protocol according to the established OLGA and OLGIM staging systems [19]. Histopathologists will be blinded to whether biopsies were directed at areas suspicious for intestinal metaplasia and to the biopsy results of WLE staging. A proportion of biopsy samples will be reviewed and re-scored by a second expert GI histopathologist, who is blinded to the initial results. This is to ensure inter-observer reproducibility for histopathological detection of intestinal metaplasia.

Serology assessment

A proportion of the collected serum will be used to assess *Helicobacter pylori (H. pylori)* serology, pepsinogen I/II ratio and gastrin-17. The remaining serum will then be stored for use in future studies exploring the development of molecular biomarkers for gastric atrophy risk stratification.

Data collection and management

All data collected for this study will be recorded in an anonymised format on a centralised, secure web-based platform (OpenClinica). Source data will be recorded in patients' notes or electronic health records, and hard copies of consent forms will be stored in a secure locked cabinet per site. All study data will be stored in a linked anonymised fashion against a study number, with the registry of study numbers stored separately on an encrypted database.

Statistical analyses

For descriptive statistics, mean (±SD) will be used in case of a normal distribution of variables and median (25%-75%) will be used for variables with a skewed distribution. Where appropriate, the Student t test or Mann-Whitney U test will be used.

Diagnostic accuracy of endoscopic diagnosis of chronic atrophic gastritis and intestinal metaplasia is defined as the total number of directed biopsies that confirm the endoscopic impression of the presence or absence of intestinal metaplasia divided by the total number

For peer review only

of biopsies (accuracy = true positives + true negatives / all biopsies). Results will be compared to the histopathology outcomes using the chi-squared test after multiple testing correction as well as kappa values for interobserver agreement among endoscopists and histopathologists.

After study completion, all videos will be collated and anonymised prior to expert panel review and estimation of the severity and extent of atrophy as well as intestinal metaplasia. 5 expert reviewers will review 50 videos each (Kappa 0.4) for the purposes of assessing inter-observer reliability. Sensitivity, specificity, and global accuracy along with the 95% confidence intervals will be established. Duration of inspection time of gastric mucosa and its relation to diagnostic accuracy will be evaluated using, when appropriate, a paired t-test or Wilcoxon test. All tests will be two-sided.

For peer review only

Discussion

The recently updated European MAPS guidelines recommend surveillance of patients with premalignant gastric mucosal lesions by performing endoscopy (preferably with advanced imaging) and taking random biopsies of the stomach for histopathological assessment. This enables the detection of progression to high-risk lesions and eventually cancer [20]. However, various studies indicate that a marked proportion of advanced gastric lesions are missed at a stage when these lesions are potentially still amenable to endoscopic management. This implies that the risk of undertreatment is undeniable [9]. The development of high-definition endoscopy and virtual chromoendoscopy has been a main focus of research in the past years and it has revolutionised the endoscopic assessment of the premalignant stomach by being superior to white light imaging. [21]. Therefore, the updated MAPS guidelines opts for the use of advanced imaging as the preferred surveillance method. Still, histological confirmation is needed through random biopsies. Future steps are to evaluate the possible shift towards an endoscopy-led strategy now these marked improvements in endoscopic technology are within our reach. This prospective study was therefore designed to determine the validity of endoscopy-led staging of the premalignant stomach using advanced imaging and taking targeted biopsies for histological confirmation.

A previous comparative study between white light and high-definition endoscopy for the diagnosis of premalignant gastric lesions indeed showed a superior diagnostic accuracy of high-definition endoscopy [15]. However, one limitation was that WLE and high-definition endoscopy were performed during one occasion, which implied that the endoscopist was not blinded to the WLE results. Within the current protocol, we choose to perform the procedures on two separate occasions with blinding of the expert endoscopist to the previous WLE results.

Over the years serological markers have shown major promise for predicting the presence and severity of gastric premalignant lesions [22, 23, 24]. Pepsinogens are serological

For peer review only

markers for atrophy in the stomach and can be divided into pepsinogen I and II. A decreased PG I/II ratio indicates the presence of atrophic changes. Gastrin serum levels are indicative for gastric acd output and are increased in the presence of atrophic changes [24]. The collection of serum samples was included in our protocol to strengthen risk stratification for progression of premalignant gastric lesions.

A few limitations of the study should be mentioned. All high-definition endoscopies will be performed by expert endoscopists at either site. A potential caveat with this design is the generalisability of the study outcomes to non-expert settings. To test this, we selected a panel of independent endoscopists who all will review recorded endoscopy videos in order to assess inter-observer variability. The same limitation holds for the histopathological evaluation of the biopsy samples. Therefore, a proportion of the samples will be reviewed and re-scored by a blinded second expert GI histopathologist.

In conclusion, prospective validation of endoscopy-led staging of the premalignant stomach will provide the needed evidence for an endoscopy-led risk stratification of patients at risk for gastric adenocarcinoma. This will allow rational design of tiered screening and surveillance protocols to benefit early-stage gastric cancer detection within at-risk populations. This will major implications for affected patients and general health care resource utilisation.

Funding statement

This work was supported by a grant from the Dutch Digestive Foundation grant number D17-22.

Competing interest statement

All authors have nothing to declare.

Contributorship

EJK and MRB conceived the idea for the study, designed the protocol and supervise study execution. MCWS, DG and MJ supervise study execution. SAVN and WW drafted the manuscript. EJK, MRB, DG, MJ, MCWS, SAVN and WW analyse and interpret data. MRJ

For peer review only

and KB interpret data. All authors provided critical revision of the manuscript for important intellectual content and approved the final draft of the protocol for submission.

For peer review only

References

1 Stomach cancer incidence statistics. 2015.

2 Global cancer statistics. World Health Organization.

3 Amiri M, Janssen F, Kunst AE. The decline in stomach cancer mortality: exploration of future trends in seven European countries. Eur J Epidemiol 2011;**26**:23-8.

4 Rahman R, Asombang AW, Ibdah JA. Characteristics of gastric cancer in Asia. World J Gastroenterol 2014;**20**:4483-90.

5 Song H, Held M, Sandin S, Rautelin H, Eliasson M, Soderberg S*, et al.* Increase in the Prevalence of Atrophic Gastritis Among Adults Age 35 to 44 Years Old in Northern Sweden Between 1990 and 2009. Clin Gastroenterol Hepatol 2015;**13**:1592-600 e1.

6 Anderson WF, Camargo MC, Fraumeni JF, Jr., Correa P, Rosenberg PS, Rabkin CS. Age-specific trends in incidence of noncardia gastric cancer in US adults. Jama 2010;**303**:1723-8.

7 Maconi G, Manes G, Porro GB. Role of symptoms in diagnosis and outcome of gastric cancer. World J Gastroenterol 2008;**14**:1149-55.

8 Matsuda T, Saika K. The 5-year relative survival rate of stomach cancer in the USA, Europe and Japan. Jpn J Clin Oncol 2013;**43**:1157-8.

9 Ren W, Yu J, Zhang ZM, Song YK, Li YH, Wang L. Missed diagnosis of early gastric cancer or high-grade intraepithelial neoplasia. World J Gastroenterol 2013;**19**:2092-6.

10 Menon S, Trudgill N. How commonly is upper gastrointestinal cancer missed at endoscopy? A meta-analysis. Endosc Int Open 2014;**2**:E46-50.

11 Dinis-Ribeiro M, Areia M, de Vries AC, Marcos-Pinto R, Monteiro-Soares M, O'Connor A*, et al.* Management of precancerous conditions and lesions in the stomach (MAPS): guideline from the European Society of Gastrointestinal Endoscopy (ESGE), European Helicobacter Study Group (EHSG), European Society of Pathology (ESP), and the Sociedade Portuguesa de Endoscopia Digestiva (SPED). Endoscopy 2012;**44**:74-94.

12 Yue H, Shan L, Bin L. The significance of OLGA and OLGIM staging systems in the risk assessment of gastric cancer: a systematic review and meta-analysis. Gastric Cancer 2018;**21**:579-87.

13 Miwata T, Quach DT, Hiyama T, Aoki R, Le HM, Tran PL*, et al.* Interobserver and intraobserver agreement for gastric mucosa atrophy. BMC Gastroenterol 2015;**15**:95.

14 Toyoshima O, Yamaji Y, Yoshida S, Matsumoto S, Yamashita H, Kanazawa T*, et al.* Endoscopic gastric atrophy is strongly associated with gastric cancer development after Helicobacter pylori eradication. Surg Endosc 2017;**31**:2140-8.

For peer review only

15 Pimentel-Nunes P, Libanio D, Lage J, Abrantes D, Coimbra M, Esposito G*, et al.* A multicenter prospective study of the real-time use of narrow-band imaging in the diagnosis of premalignant gastric conditions and lesions. Endoscopy 2016;**48**:723-30.

16 Xirouchakis E, Laoudi F, Tsartsali L, Spiliadi C, Georgopoulos SD. Screening for gastric premalignant lesions with narrow band imaging, white light and updated Sydney protocol or both? Dig Dis Sci 2013;**58**:1084-90.

17 Uedo N, Ishihara R, Iishi H, Yamamoto S, Yamamoto S, Yamada T*, et al.* A new method of diagnosing gastric intestinal metaplasia: narrow-band imaging with magnifying endoscopy. Endoscopy 2006;**38**:819-24.

18 Dixon MF, Genta RM, Yardley JH, Correa P. Classification and grading of gastritis. The updated Sydney System. International Workshop on the Histopathology of Gastritis, Houston 1994. Am J Surg Pathol 1996;**20**:1161-81.

19 Cho SJ, Choi IJ, Kook MC, Nam BH, Kim CG, Lee JY*, et al.* Staging of intestinal- and diffuse-type gastric cancers with the OLGA and OLGIM staging systems. Aliment Pharmacol Ther 2013;**38**:1292-302.

20 Pimentel-Nunes P, Libanio D, Marcos-Pinto R, Areia M, Leja M, Esposito G*, et al.* Management of epithelial precancerous conditions and lesions in the stomach (MAPS II): European Society of Gastrointestinal Endoscopy (ESGE), European Helicobacter and Microbiota Study Group (EHMSG), European Society of Pathology (ESP), and Sociedade Portuguesa de Endoscopia Digestiva (SPED) guideline update 2019. Endoscopy 2019.

21 East JE, Vleugels JL, Roelandt P, Bhandari P, Bisschops R, Dekker E*, et al.* Advanced endoscopic imaging: European Society of Gastrointestinal Endoscopy (ESGE) Technology Review. Endoscopy 2016;**48**:1029-45.

22 den Hollander WJ, Holster IL, den Hoed CM, Capelle LG, Tang TJ, Anten MP*, et al.* Surveillance of premalignant gastric lesions: a multicentre prospective cohort study from low incidence regions. Gut 2018.

23 Huang YK, Yu JC, Kang WM, Ma ZQ, Ye X, Tian SB*, et al.* Significance of Serum Pepsinogens as a Biomarker for Gastric Cancer and Atrophic Gastritis Screening: A Systematic Review and Meta-Analysis. PLoS One 2015;**10**:e0142080.

24 Leja M, Kupcinskas L, Funka K, Sudraba A, Jonaitis L, Ivanauskas A*, et al.* Value of gastrin-17 in detecting antral atrophy. Adv Med Sci 2011;**56**:145-50.

For peer review only

Figure 1 Flowchart of the study design; *CAG* chronic atrophic gastritis, *EGGIM* endoscopic grading of gastric intestinal metaplasia *GI* gastrointestinal, *H. pylori* Helicobacter pylori, *OLGA* operative link for gastritis assessment, *OLGIM* operative link for gastric intestinal metaplasia assessment, *WLE* white light endoscopy

Figure 2 Biopsy strategy. *A.) Sydney protocol biopsy sites in the opened stomach along the greater curvature; B.) Biopsy sites in the anatomical view.*

For peer review only

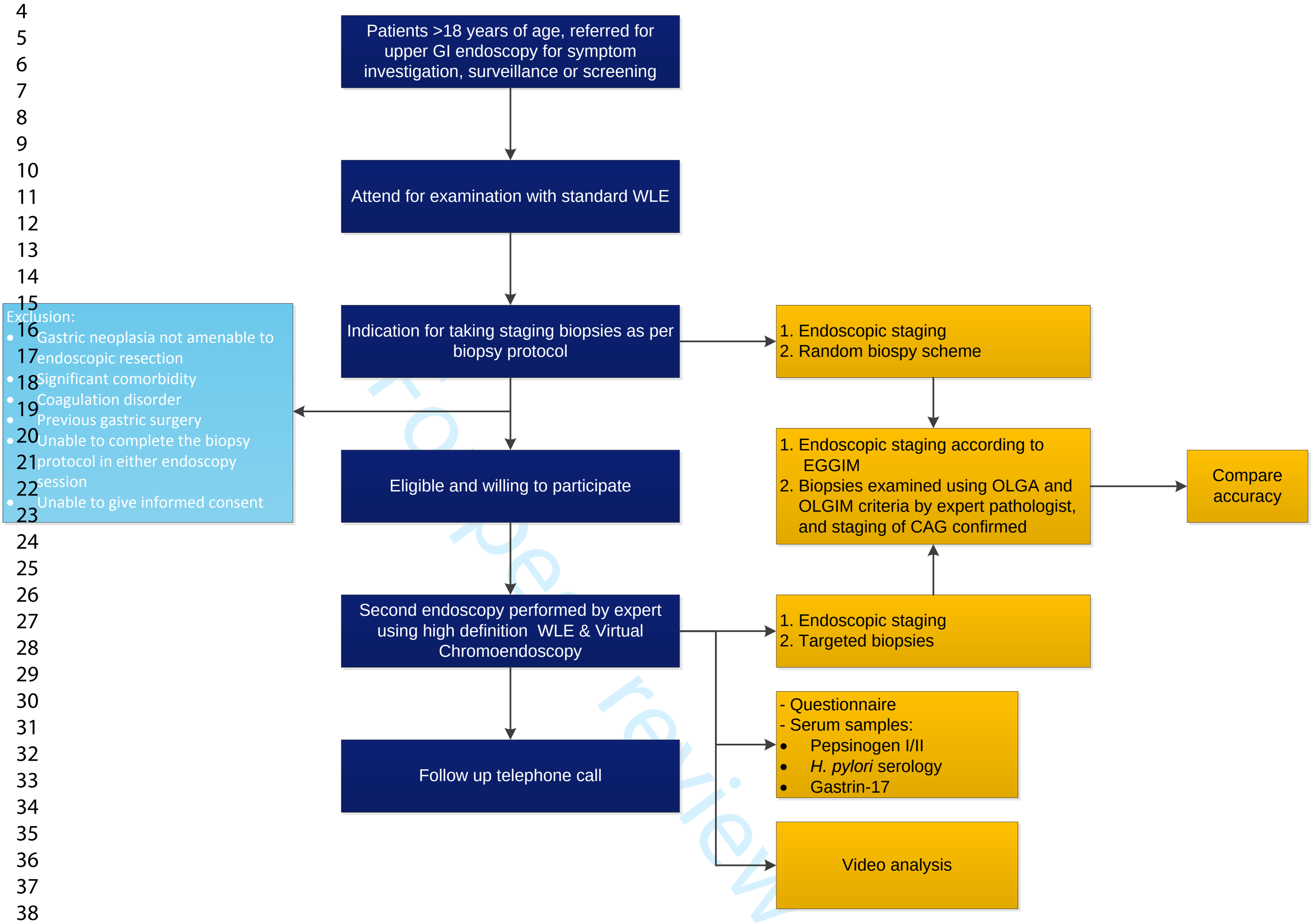

Figure 1 Flowchart of the study design; *CAG* chronic atrophic gastritis, *EGGIM* endoscopic grading of gastric intestinal metaplasia *GI* gastrointestinal, *H. pylori* Helicobacter pylori, *OLGA* operative link for gastritis assessment, *OLGIM* operative link for gastric intestinal metaplasia assessment, *WLE* white light endoscopy

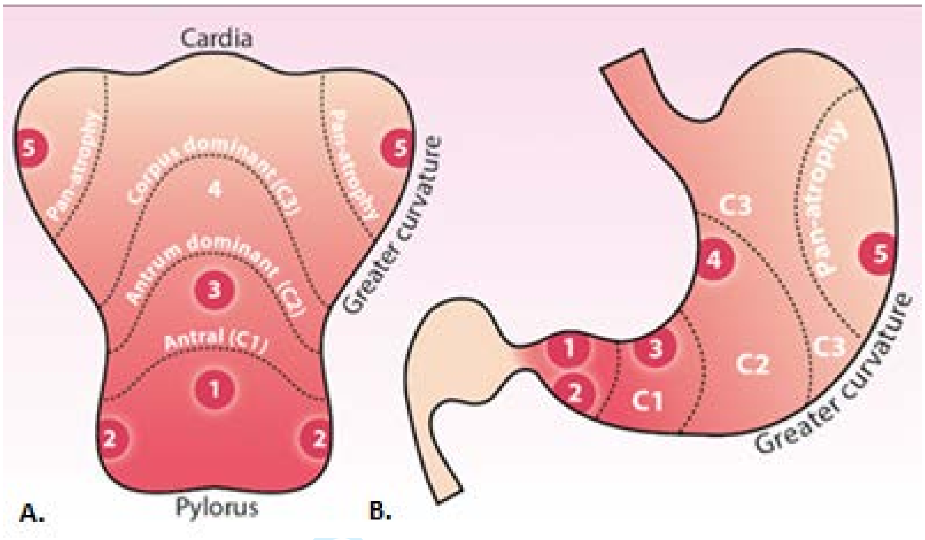

Figure 2 Biopsy strategy. *A.) Sydney protocol biopsy sites in the opened stomach along the greater curvature; B.) Biopsy sites in the anatomical view.*

Section & Topic	No	Item	Reported on page #
**TITLE OR ABSTRACT**			
	**1**	Identification as a study of diagnostic accuracy using at least one measure of accuracy (such as sensitivity, specificity, predictive values, or AUC)	1
**ABSTRACT**			
	**2**	Structured summary of study design, methods, results, and conclusions (for specific guidance, see STARD for Abstracts)	2
**INTRODUCTION**			
	**3**	Scientific and clinical background, including the intended use and clinical role of the index test	4
	**4**	Study objectives and hypotheses	6
**METHODS**			
Study design	**5**	Whether data collection was planned before the index test and reference standard were performed (prospective study) or after (retrospective study)	6
Participants	**6**	Eligibility criteria	7
	**7**	On what basis potentially eligible participants were identified (such as symptoms, results from previous tests, inclusion in registry)	7
	**8**	Where and when potentially eligible participants were identified (setting, location and dates)	7
	**9**	Whether participants formed a consecutive, random or convenience series	7
Test methods	**10a**	Index test, in sufficient detail to allow replication	8
	**10b**	Reference standard, in sufficient detail to allow replication	8
	**11**	Rationale for choosing the reference standard (if alternatives exist)	4
	**12a**	Definition of and rationale for test positivity cut-offs or result categories of the index test, distinguishing pre-specified from exploratory	9
	**12b**	Definition of and rationale for test positivity cut-offs or result categories of the reference standard, distinguishing pre-specified from exploratory	9
	**13a**	Whether clinical information and reference standard results were available to the performers/readers of the index test	4
	**13b**	Whether clinical information and index test results were available to the assessors of the reference standard	4
Analysis	**14**	Methods for estimating or comparing measures of diagnostic accuracy	7-9
	**15**	How indeterminate index test or reference standard results were handled	9
	**16**	How missing data on the index test and reference standard were handled	9
	**17**	Any analyses of variability in diagnostic accuracy, distinguishing pre-specified from exploratory	9
	**18**	Intended sample size and how it was determined	9
**RESULTS**			
Participants	**19**	Flow of participants, using a diagram	Fig 1
	**20**	Baseline demographic and clinical characteristics of participants	Protocol, i.e. no results provided yet
	**21a**	Distribution of severity of disease in those with the target condition	Protocol, i.e. no results provided yet
	**21b**	Distribution of alternative diagnoses in those without the target condition	Protocol, i.e. no results provided yet
	**22**	Time interval and any clinical interventions between index test and reference standard	Protocol, i.e. no results provided yet
Test results	**23**	Cross tabulation of the index test results (or their distribution) by the results of the reference standard	Protocol, i.e. no results provided yet
	**24**	Estimates of diagnostic accuracy and their precision (such as 95% confidence intervals)	Protocol, i.e. no results provided yet
	**25**	Any adverse events from performing the index test or the reference standard	Protocol, i.e. no results provided yet
**DISCUSSION**			
	**26**	Study limitations, including sources of potential bias, statistical uncertainty, and generalisability	11
	**27**	Implications for practice, including the intended use and clinical role of the index test	11
**OTHER INFORMATION**			

For peer review only

CC BY SA

	**28**	Registration number and name of registry	2
	**29**	Where the full study protocol can be accessed	2
	**30**	Sources of funding and other support; role of funders	11

For peer review only

STARD 2015

AIM

STARD stands for "Standards for Reporting Diagnostic accuracy studies". This list of items was developed to contribute to the completeness and transparency of reporting of diagnostic accuracy studies. Authors can use the list to write informative study reports. Editors and peer-reviewers can use it to evaluate whether the information has been included in manuscripts submitted for publication.

EXPLANATION

A **diagnostic accuracy study** evaluates the ability of one or more medical tests to correctly classify study participants as having a **target condition.** This can be a disease, a disease stage, response or benefit from therapy, or an event or condition in the future. A medical test can be an imaging procedure, a laboratory test, elements from history and physical examination, a combination of these, or any other method for collecting information about the current health status of a patient.

The test whose accuracy is evaluated is called **index test.** A study can evaluate the accuracy of one or more index tests. Evaluating the ability of a medical test to correctly classify patients is typically done by comparing the distribution of the index test results with those of the **reference standard**. The reference standard is the best available method for establishing the presence or absence of the target condition. An accuracy study can rely on one or more reference standards.

If test results are categorized as either positive or negative, the cross tabulation of the index test results against those of the reference standard can be used to estimate the **sensitivity** of the index test (the proportion of participants *with* the target condition who have a positive index test), and its **specificity** (the proportion *without* the target condition who have a negative index test). From this cross tabulation (sometimes referred to as the contingency or "2x2" table), several other accuracy statistics can be estimated, such as the positive and negative **predictive values** of the test. Confidence intervals around estimates of accuracy can then be calculated to quantify the statistical **precision** of the measurements.

If the index test results can take more than two values, categorization of test results as positive or negative requires a **test positivity cut-off**. When multiple such cut-offs can be defined, authors can report a receiver operating characteristic (ROC) curve which graphically represents the combination of sensitivity and specificity for each possible test positivity cut-off. The **area under the ROC curve** informs in a single numerical value about the overall diagnostic accuracy of the index test.

The **intended use** of a medical test can be diagnosis, screening, staging, monitoring, surveillance, prediction or prognosis. The **clinical role** of a test explains its position relative to existing tests in the clinical pathway. A replacement test, for example, replaces an existing test. A triage test is used before an existing test; an add-on test is used after an existing test.

Besides diagnostic accuracy, several other outcomes and statistics may be relevant in the evaluation of medical tests. Medical tests can also be used to classify patients for purposes other than diagnosis, such as staging or prognosis. The STARD list was not explicitly developed for these other outcomes, statistics, and study types, although most STARD items would still apply.

DEVELOPMENT

This STARD list was released in 2015. The 30 items were identified by an international expert group of methodologists, researchers, and editors. The guiding principle in the development of STARD was to select items that, when reported, would help readers to judge the potential for bias in the study, to appraise the applicability of the study findings and the validity of conclusions and recommendations. The list represents an update of the first version, which was published in 2003.

More information can be found on http://www.equator-network.org/reporting-guidelines/stard.

CC BY SA

For peer review only - http://bmjopen.bmj.com/site/about/guidelines.xhtml

BMJ Open

ACCURACY OF ENDOSCOPIC STAGING AND TARGETED BIOPSIES FOR ROUTINE GASTRIC INTESTINAL METAPLASIA AND GASTRIC ATROPHY EVALUATION: STUDY PROTOCOL OF A PROSPECTIVE, COHORT STUDY – THE ESTIMATE STUDY -

Journal:	*BMJ Open*
Manuscript ID	bmjopen-2019-032013.R1
Article Type:	Protocol
Date Submitted by the Author:	31-Jul-2019
Complete List of Authors:	Nieuwenburg, Stella; Erasmus MC University Medical Center, Gastroenterology & Hepatology<br>Waddingham, William; University College London Hospital, Endoscopy; UCL Cancer Institute<br>Graham, David; University College London Hospital, Endoscopy<br>Rodriguez-Justo, Manuel; University College London Hospital, Pathology<br>Biermann, Katharina; Erasmus MC University Medical Center, Pathology<br>Kuipers, Ernst; Erasmus MC University Medical Center, Gastroenterology & Hepatology<br>Banks, Matthew; University College London Hospital, Endoscopy<br>Jansen, Marnix; University College London Hospital, Endoscopy; University College London Hospital, Pathology<br>Spaander, Manon; Erasmus MC University Medical Center, Gastroenterology &Hepatology
**Primary Subject Heading**:	Gastroenterology and hepatology
Secondary Subject Heading:	Oncology
Keywords:	Endoscopy < GASTROENTEROLOGY, Gastrointestinal tumours < GASTROENTEROLOGY, PREVENTIVE MEDICINE

SCHOLARONE™
Manuscripts

ACCURACY OF ENDOSCOPIC STAGING AND TARGETED BIOPSIES FOR ROUTINE GASTRIC INTESTINAL METAPLASIA AND GASTRIC ATROPHY EVALUATION STUDY PROTOCOL OF A PROSPECTIVE, COHORT STUDY – THE ESTIMATE STUDY -

Stella A.V. Nieuwenburg[1]*, William W. Waddingham[2,3]*, David Graham[2], Manuel Rodriguez-Justo[3,4], Katharina Biermann[5], Ernst J. Kuipers[1], Matthew R. Banks[2], Marnix Jansen[2,4], Manon C.W. Spaander[1]

** Authors share first authorship*

[1] *Department of Gastroenterology and Hepatology, Erasmus MC University Medical Center, Rotterdam, the Netherlands*

[2] *Department of Endoscopy, University College London Hospital, London, UK*

[3] *UCL Cancer Institute, University College London, London, UK*

[4] *Department of Pathology, University College London, London, UK*

[5] *Department of Pathology, Erasmus MC University Medical Center, Rotterdam, The Netherlands*

CORRESPONDENCE:

Manon C.W. Spaander, MD, PhD, Associate professor

Department of Gastroenterology and Hepatology (room Hs-308)

Erasmus MC University Medical Center

‘s Gravendijkwal 230, 3015 CE Rotterdam, The Netherlands

Telephone: +31 (0) 10 7035643 Fax: +31 (0) 10 7035172

E-mail: v.spaander@erasmusmc.nl

For peer review only

Abstract

Introduction Patients with chronic atrophic gastritis (CAG) and intestinal metaplasia (IM) are at risk of developing gastric adenocarcinoma. Their diagnosis and management currently relies on histopathological guidance after random endoscopic biopsy sampling (Sydney biopsy strategy). This approach has significant flaws such as under-diagnosis, poor reproducibility and poor correlation between endoscopy and histology. This prospective, international multicentre study aims to establish whether endoscopy-led risk stratification accurately and reproducibly predicts CAG and IM extent and disease stage.

Methods and analysis Patients with CAG and/or IM on standard white light endoscopy (WLE) will be prospectively identified and invited to undergo a second endoscopy performed by an expert endoscopist using enhanced endoscopic imaging techniques with virtual chromoendoscopy. Extent of CAG/IM will be endoscopically staged with enhanced imaging and compared to standard WLE. Histopathologic risk stratification through targeted biopsies will be compared to endoscopic disease staging and to random biopsy staging on WLE as a reference. At least 234 patients are required to show a 10% difference in sensitivity and accuracy between enhanced imaging endoscopy-led staging and the current biopsy-led staging protocol of gastric atrophy with a power (beta) of 80% and a 0.05 probability of a type I error (alpha).

Ethics and dissemination The study was approved by the respective Institutional Review Boards (Netherlands: MEC-2018-078; UK: 19/LO/0089). The findings will be published in peer-reviewed journals and presented at scientific meetings.

Trial registration number NTC=7661

For peer review only

Strengths and limitations of this study

- This is the first study to compare endoscopy-led risk stratification of premalignant gastric lesions using advanced imaging and targeted biopsies with white light endoscopy and random biopsies as a reference, performed at separate occasions with the endoscopist blinded to prior results
- This study will additionally provide biobank biopsy and serum material for future biomarker analysis
- A possible limitation of this study is that all procedures will be performed by expert endoscopists in teaching hospitals. Endoscopy-led staging should be feasible outside of expert settings to deliver actual changes in current practice. External reproducibility will be evaluated using interobserver variability of disease staging through video recordings
- The same limitation holds for the histopathological evaluation of the biopsy samples. Therefore, a proportion of the samples will be reviewed and re-scored by a blinded second expert GI histopathologist.

For peer review only

Introduction

Gastric adenocarcinoma remains a major cause of cancer mortality and is the most commonly diagnosed malignant condition of the gastrointestinal tract (1-4). Although incidence rates had previously been declining, recent studies demonstrated this differs among population subgroups. For example, an increasing incidence of gastric adenocarcinoma among young white cohorts in Western countries was objectified. This may be due to an increasing prevalence of gastric cancer precursors among younger adults, in particular chronic atrophic gastritis (CAG),intestinal metaplasia (IM), and dysplasia (5, 6). These studies suggest that gastric cancer incidence rates may plateau or even increase again in the upcoming years. Importantly, with the exception of Japan and Korea, the majority of gastric cancers worldwide are diagnosed at a late stage. This results in a poor prognosis with a less than 30% 5-year survival (1, 2, 7). Japan's earlier stage of diagnosis and superior 5-year survival highlight the need for earlier recognition and treatment (8).

Endoscopic recognition of the premalignant stomach has long been problematic and limited by the ability of endoscopist and the imaging tools. A previous study demonstrated that 22% of high-grade dysplastic (HGD) lesions and early gastric cancers were missed (9, 10). A meta-analysis and systematic review of endoscopy follow-up studies confirmed that a marked proportion of early gastric cancers are missed at endoscopy (10). Therefore, current practice uses histology based staging (11, 12). However, endoscopic imaging has significantly improved with high-definition endoscopes and imaging enhancement technologies now routinely available. Some recent studies already suggested that accurate endoscopic staging of CAG and GIM is achievable and can robustly predict gastric adenocarcinoma risk. Importantly, the interobserver and intraobserver reproducibility characteristics of endoscopic CAG and GIM severity assessment are in experienced hands moderate to excellent (13-18). These marked improvements in endoscopic technology and the shift towards an endoscopy-led approach will empower the endoscopist to risk stratify individuals with greater accuracy and decrease the already huge burden placed on our

For peer review only

endoscopy and histopathology departments. Therefore, the aim of this study is to evaluate if enhanced endoscopic imaging, including high-definition white light endoscopy and virtual chromoendoscopy, alongside targeted biopsies, provides an accurate and reproducible assessment of CAG and IM disease extent and staging, when compared to the current practice of white light endoscopy and random biopsies through the Sydney protocol biopsy strategy.

For peer review only

Methods and analysis

AIMS

The primary aim of this study is to assess the diagnostic accuracy for the endoscopic diagnosis of intestinal metaplasia in Sydney biopsy locations comparing standard endoscopic staging with random biopsies to enhanced imaging with biopsies targeted to GIM (19). The Standards for Reporting of Diagnostic Accuracy Studies (STARD) guidelines were followed(20). Study sites are located in the Netherlands and the United Kingdom. Secondary objectives are to evaluate a) reproducibility of endoscopic staging after expert review, b) reproducibility of histopathology for detection of intestinal metaplasia, c) the number of dysplastic or neoplastic lesions detected, and d) effects of inspection time of gastric mucosa on diagnostic accuracy.

DESIGN

This is a prospective, multicentre registry study on the accuracy and reproducibility of enhanced endoscopic imaging, including high-definition white light endoscopy and virtual chromoendoscopy, for the staging of chronic atrophic gastritis and intestinal metaplasia. Two upper endoscopies will be performed on two separate occasions (6-12 months in between) using standard white-light endoscopy plus random biopsies (current diagnostic strategy) at the first endoscopy and enhanced endoscopic imaging with targeted biopsies (proposed diagnostic strategy) at the second endoscopy. We will compare both approaches using histopathology as a reference and assess the accuracy and reproducibility of enhanced endoscopic imaging (**Fig 1**). The study protocol was approved by respective Institutional Review Boards (Netherlands: MEC-2018-078; UK: 19/LO/0089). The protocol has been registered in the Dutch trial register (NTR=7661).

For peer review only

PATIENT AND PUBLIC INVOLVEMENT

We maintained close links with patient alliances and interest groups, both in the Netherlands as well as in the UK. This close relationship informs our practice and is the basis for the current study design. We will engage closely with patient interest groups to communicate research findings and ensure that our deliverables are fit for purpose.

PARTICIPANTS

Sample size

For estimation of sample size we assume that the diagnosis of CAG or IM on enhanced imaging and targeted biopsies must be set with at least a 90% sensitivity with white light endoscopy and random biopsies as a reference (15). A power (beta) of 80% and a probability of type I error (alpha) of 0.05 will be handled. That purpose requires at least 234 patients to be recruited to show a 10% difference in sensitivity between enhanced endoscopy-led staging and the standard WLE.

Recruitment

All patients (> 18 years of age) referred to the endoscopy department for routine diagnostic upper gastrointestinal endoscopy and diagnosed with chronic atrophic gastritis or intestinal metaplasia between November 2018 and June 2020 are eligible for inclusion if able to give informed consent. Patients are excluded when having 1) gastric neoplasia not amenable to endoscopic resection, 2) no indication for Sydney biopsy staging on standard WLE, 3) significant comorbidity, 4) a coagulation disorder, 5) previous gastric surgery, or 6) are unable to complete the biopsy protocol in either endoscopy session.

INTERVENTIONS

Baseline characteristics

All patients are asked to complete a questionnaire on lifestyle factors, medical history, past interventions, medication use and family history of gastric cancer.

For peer review only

White light endoscopy

Patients referred to the endoscopy department for upper gastrointestinal endoscopy for investigation of symptoms or for surveillance of a known condition will undergo their procedure on a standard diagnostic gastroscopy list. Patients found to have chronic atrophic gastritis or intestinal metaplasia will be prospectively identified. During the initial procedure patients will receive the current recommended practice. Current practice is to initially identify if gastric atrophy is present and to inspect the gastric mucosa for areas suspicious for dysplasia or malignancy. Any mucosal abnormalities suspicious for dysplasia or malignancy are biopsied with tissue biopsies placed in separate containers. Following this, ten random biopsies are taken according to the Sydney protocol (see also **Fig. 2**): 4 quadrant biopsies of the antrum, 2 biopsies from the incisura, and 4 biopsies from the body of the stomach, respectively two from the lesser curve, and two from the greater curve.

Enhanced imaging endoscopy

Patients who opt to be recruited to the study will be invited for a second endoscopy at 6 to 12-months interval. This will be performed by one of the experts on this protocol. The endoscopists will be blinded to any previous endoscopy or biopsy results. This second endoscopy will be recorded and performed using enhanced endoscopic imaging. Given that these patients will have recently undergone a complete upper gastrointestinal endoscopy, whilst all anatomical landmarks will be viewed, the focus of this examination will be on the gastric mucosa. The endoscopist will record 1) the extent of gastric atrophy, 2) the presence and extent of intestinal metaplasia in each of the aforementioned areas. This will be done using our simplified endoscopic metaplasia scoring system (GRAHAM score) (**Table 1**). Biopsies will then be taken in the following manner: 1) Areas of intestinal metaplasia found in any of the Sydney protocol areas, 2) Sydney areas negative for gastric intestinal metaplasia will be randomly biopsied, as control, to complete the assessment, 3) Lesions suspicious for dysplasia or malignancy.

For peer review only

	Focal / Minimal metaplasia (<1/3 of surface coverage)	Moderate / extensive metaplasia (>1/3 surface coverage)
Antrum & incisura	1	2
Lesser curve	1	2
Greater curve	1	2

Table 1 Simplified endoscopic gastric intestinal metaplasia staging system: "GRAHAM Score"

Histopathological assessment

Each biopsy will be reviewed at the teaching hospital by one of the expert gastrointestinal histopathologists named on this protocol according to the established OLGA and OLGIM staging systems (21). Histopathologists will be blinded to whether biopsies were directed at areas suspicious for intestinal metaplasia and to the biopsy results of WLE staging. A proportion of biopsy samples will be reviewed and re-scored by a second expert GI histopathologist, who is blinded to the initial results. This is to ensure inter-observer reproducibility for histopathological detection of intestinal metaplasia.

Serology assessment

A proportion of the collected serum will be used to assess *Helicobacter pylori (H. pylori)* serology, pepsinogen I/II ratio and gastrin-17. The remaining serum will then be stored for use in future studies exploring the development of molecular biomarkers for gastric atrophy risk stratification.

Data collection and management

All data collected for this study will be recorded in an anonymised format on a centralised, secure web-based platform (OpenClinica). Source data will be recorded in patients' notes or electronic health records, and hard copies of consent forms will be stored in a secure locked cabinet per site. All study data will be stored in a linked anonymised fashion against a study number, with the registry of study numbers stored separately on an encrypted database.

For peer review only

Statistical analyses

For descriptive statistics, mean (±SD) will be used in case of a normal distribution of variables and median (25%-75%) will be used for variables with a skewed distribution. Where appropriate, the Student t test or Mann-Whitney U test will be used.

Diagnostic accuracy of endoscopic diagnosis of chronic atrophic gastritis and intestinal metaplasia is defined as the total number of directed biopsies that confirm the endoscopic impression of the presence or absence of intestinal metaplasia divided by the total number of biopsies (accuracy = true positives + true negatives / all biopsies). Results will be compared to the histopathology outcomes using the chi-squared test after multiple testing correction as well as kappa values for interobserver agreement among endoscopists and histopathologists.

After study completion, all videos will be collated and anonymised prior to expert panel review and estimation of the severity and extent of atrophy as well as intestinal metaplasia. 5 expert reviewers will review 50 videos each (Kappa 0.4) for the purposes of assessing inter-observer reliability. Sensitivity, specificity, and global accuracy along with the 95% confidence intervals will be established. Duration of inspection time of gastric mucosa and its relation to diagnostic accuracy will be evaluated using, when appropriate, a paired t-test or Wilcoxon test. All tests will be two-sided.

For peer review only

Discussion

The recently updated European MAPS guidelines recommend surveillance of patients with premalignant gastric mucosal lesions by performing endoscopy (preferably with advanced imaging) and taking random biopsies of the stomach for histopathological assessment. This enables the detection of progression to high-risk lesions and eventually cancer (22). However, various studies indicate that a marked proportion of advanced gastric lesions are missed at a stage when these lesions are potentially still amenable to endoscopic management. This implies that the risk of undertreatment is undeniable (9). The development of high-definition endoscopy and virtual chromoendoscopy has been a main focus of research in the past years and it has revolutionised the endoscopic assessment of the premalignant stomach by being superior to white light imaging. (23). The updated MAPS guidelines opts for the use of advanced imaging as the preferred surveillance method. Recently, Esposito G et al showed a scoring tool based on endoscopic staging using Endoscopic Grading of Gastric Intestinal Metaplasia (EGGIM) with advanced imaging as a promising decision tool to identify patients at risk of gastric cancer (24). However, currently there are no studies on how the use of advanced endoscopic imaging to detect IM of the stomach can be applied in countries with a low prevalence of IM. Still, histological confirmation is needed through random biopsies. Future steps are to evaluate the possible shift towards an endoscopy-led strategy now these marked improvements in endoscopic technology are within our reach. This prospective study was therefore designed to determine the validity of endoscopy-led staging of the premalignant stomach using advanced imaging and taking targeted biopsies for histological confirmation.

A previous comparative study between white light and high-definition endoscopy for the diagnosis of premalignant gastric lesions indeed showed a superior diagnostic accuracy of high-definition endoscopy (15). However, one limitation was that WLE and high-definition endoscopy were performed during one occasion, which implied that the endoscopist was not blinded to the WLE results. Within the current protocol, we choose to perform the procedures

For peer review only

on two separate occasions with blinding of the expert endoscopist to the previous WLE results.

Over the years serological markers have shown major promise for predicting the presence and severity of gastric premalignant lesions (25-27). Pepsinogens are serological markers for atrophy in the stomach and can be divided into pepsinogen I and II. A decreased PG I/II ratio indicates the presence of atrophic changes. Gastrin serum levels are indicative for gastric acd output and are increased in the presence of atrophic changes (27). The collection of serum samples was included in our protocol to strengthen risk stratification for progression of premalignant gastric lesions.

A few limitations of the study should be mentioned. All high-definition endoscopies will be performed by expert endoscopists at either site. A potential caveat with this design is the generalisability of the study outcomes to non-expert settings. To test this, we selected a panel of independent endoscopists who all will review recorded endoscopy videos in order to assess inter-observer variability. The same limitation holds for the histopathological evaluation of the biopsy samples. Therefore, a proportion of the samples will be reviewed and re-scored by a blinded second expert GI histopathologist.

In conclusion, prospective validation of endoscopy-led staging of the premalignant stomach will provide the needed evidence for an endoscopy-led risk stratification of patients at risk for gastric adenocarcinoma. This will allow rational design of tiered screening and surveillance protocols to benefit early-stage gastric cancer detection within at-risk populations. This will major implications for affected patients and general health care resource utilisation.

Funding statement

This work was supported by a grant from the Maag Lever Darm Stichting, Dutch Digestive Foundation grant number D17-22.

Competing interest statement

None declared.

For peer review only

Contributorship

EJK and MRB conceived the idea for the study, designed the protocol and supervise study execution. MCWS, DG and MJ supervise study execution. SAVN and WW drafted the manuscript. EJK, MRB, DG, MJ, MCWS, SAVN and WW analyse and interpret data. MRJ and KB interpret data. All authors provided critical revision of the manuscript for important intellectual content and approved the final draft of the protocol for submission.

For peer review only

References

1. Stomach cancer incidence statistics 2015 [Available from: http://www.cancerresearchuk.org/cancer-info/cancerstats.

2. Global cancer statistics: World Health Organization; [Available from: https://www.who.int/cancer/en/.

3. Amiri M, Janssen F, Kunst AE. The decline in stomach cancer mortality: exploration of future trends in seven European countries. Eur J Epidemiol. 2011;26(1):23-8.

4. Rahman R, Asombang AW, Ibdah JA. Characteristics of gastric cancer in Asia. World J Gastroenterol. 2014;20(16):4483-90.

5. Song H, Held M, Sandin S, Rautelin H, Eliasson M, Soderberg S, et al. Increase in the Prevalence of Atrophic Gastritis Among Adults Age 35 to 44 Years Old in Northern Sweden Between 1990 and 2009. Clin Gastroenterol Hepatol. 2015;13(9):1592-600 e1.

6. Anderson WF, Camargo MC, Fraumeni JF, Jr., Correa P, Rosenberg PS, Rabkin CS. Age-specific trends in incidence of noncardia gastric cancer in US adults. Jama. 2010;303(17):1723-8.

7. Maconi G, Manes G, Porro GB. Role of symptoms in diagnosis and outcome of gastric cancer. World J Gastroenterol. 2008;14(8):1149-55.

8. Matsuda T, Saika K. The 5-year relative survival rate of stomach cancer in the USA, Europe and Japan. Jpn J Clin Oncol. 2013;43(11):1157-8.

9. Ren W, Yu J, Zhang ZM, Song YK, Li YH, Wang L. Missed diagnosis of early gastric cancer or high-grade intraepithelial neoplasia. World J Gastroenterol. 2013;19(13):2092-6.

10. Menon S, Trudgill N. How commonly is upper gastrointestinal cancer missed at endoscopy? A meta-analysis. Endosc Int Open. 2014;2(2):E46-50.

11. Dinis-Ribeiro M, Areia M, de Vries AC, Marcos-Pinto R, Monteiro-Soares M, O'Connor A, et al. Management of precancerous conditions and lesions in the stomach (MAPS): guideline from the European Society of Gastrointestinal Endoscopy (ESGE), European Helicobacter Study Group (EHSG), European Society of Pathology (ESP), and the Sociedade Portuguesa de Endoscopia Digestiva (SPED). Endoscopy. 2012;44(1):74-94.

12. Yue H, Shan L, Bin L. The significance of OLGA and OLGIM staging systems in the risk assessment of gastric cancer: a systematic review and meta-analysis. Gastric Cancer. 2018;21(4):579-87.

13. Miwata T, Quach DT, Hiyama T, Aoki R, Le HM, Tran PL, et al. Interobserver and intraobserver agreement for gastric mucosa atrophy. BMC Gastroenterol. 2015;15:95.

14. Toyoshima O, Yamaji Y, Yoshida S, Matsumoto S, Yamashita H, Kanazawa T, et al. Endoscopic gastric atrophy is strongly associated with gastric cancer development after Helicobacter pylori eradication. Surg Endosc. 2017;31(5):2140-8.

For peer review only

15. Pimentel-Nunes P, Libanio D, Lage J, Abrantes D, Coimbra M, Esposito G, et al. A multicenter prospective study of the real-time use of narrow-band imaging in the diagnosis of premalignant gastric conditions and lesions. Endoscopy. 2016;48(8):723-30.

16. Xirouchakis E, Laoudi F, Tsartsali L, Spiliadi C, Georgopoulos SD. Screening for gastric premalignant lesions with narrow band imaging, white light and updated Sydney protocol or both? Dig Dis Sci. 2013;58(4):1084-90.

17. Uedo N, Ishihara R, Iishi H, Yamamoto S, Yamamoto S, Yamada T, et al. A new method of diagnosing gastric intestinal metaplasia: narrow-band imaging with magnifying endoscopy. Endoscopy. 2006;38(8):819-24.

18. Banks M, Graham D, Jansen M, Gotoda T, Coda S, di Pietro M, et al. British Society of Gastroenterology guidelines on the diagnosis and management of patients at risk of gastric adenocarcinoma. Gut. 2019.

19. Dixon MF, Genta RM, Yardley JH, Correa P. Classification and grading of gastritis. The updated Sydney System. International Workshop on the Histopathology of Gastritis, Houston 1994. Am J Surg Pathol. 1996;20(10):1161-81.

20. Cohen JF, Korevaar DA, Altman DG, Bruns DE, Gatsonis CA, Hooft L, et al. STARD 2015 guidelines for reporting diagnostic accuracy studies: explanation and elaboration. BMJ Open. 2016;6(11):e012799.

21. Cho SJ, Choi IJ, Kook MC, Nam BH, Kim CG, Lee JY, et al. Staging of intestinal- and diffuse-type gastric cancers with the OLGA and OLGIM staging systems. Aliment Pharmacol Ther. 2013;38(10):1292-302.

22. Pimentel-Nunes P, Libanio D, Marcos-Pinto R, Areia M, Leja M, Esposito G, et al. Management of epithelial precancerous conditions and lesions in the stomach (MAPS II): European Society of Gastrointestinal Endoscopy (ESGE), European Helicobacter and Microbiota Study Group (EHMSG), European Society of Pathology (ESP), and Sociedade Portuguesa de Endoscopia Digestiva (SPED) guideline update 2019. Endoscopy. 2019.

23. East JE, Vleugels JL, Roelandt P, Bhandari P, Bisschops R, Dekker E, et al. Advanced endoscopic imaging: European Society of Gastrointestinal Endoscopy (ESGE) Technology Review. Endoscopy. 2016;48(11):1029-45.

24. Esposito G, Pimentel-Nunes P, Angeletti S, Castro R, Libanio D, Galli G, et al. Endoscopic grading of gastric intestinal metaplasia (EGGIM): a multicenter validation study. Endoscopy. 2019;51(6):515-21.

25. den Hollander WJ, Holster IL, den Hoed CM, Capelle LG, Tang TJ, Anten MP, et al. Surveillance of premalignant gastric lesions: a multicentre prospective cohort study from low incidence regions. Gut. 2018.

For peer review only

26. Huang YK, Yu JC, Kang WM, Ma ZQ, Ye X, Tian SB, et al. Significance of Serum Pepsinogens as a Biomarker for Gastric Cancer and Atrophic Gastritis Screening: A Systematic Review and Meta-Analysis. PLoS One. 2015;10(11):e0142080.

27. Leja M, Kupcinskas L, Funka K, Sudraba A, Jonaitis L, Ivanauskas A, et al. Value of gastrin-17 in detecting antral atrophy. Adv Med Sci. 2011;56(2):145-50.

For peer review only

Figure 1 Flowchart of the study design; *CAG* chronic atrophic gastritis, *EGGIM* endoscopic grading of gastric intestinal metaplasia *GI* gastrointestinal, *H. pylori* Helicobacter pylori, *OLGA* operative link for gastritis assessment, *OLGIM* operative link for gastric intestinal metaplasia assessment, *WLE* white light endoscopy

Figure 2 Biopsy strategy. *A.) Sydney protocol biopsy sites in the opened stomach along the greater curvature; B.) Biopsy sites in the anatomical view.*

For peer review only

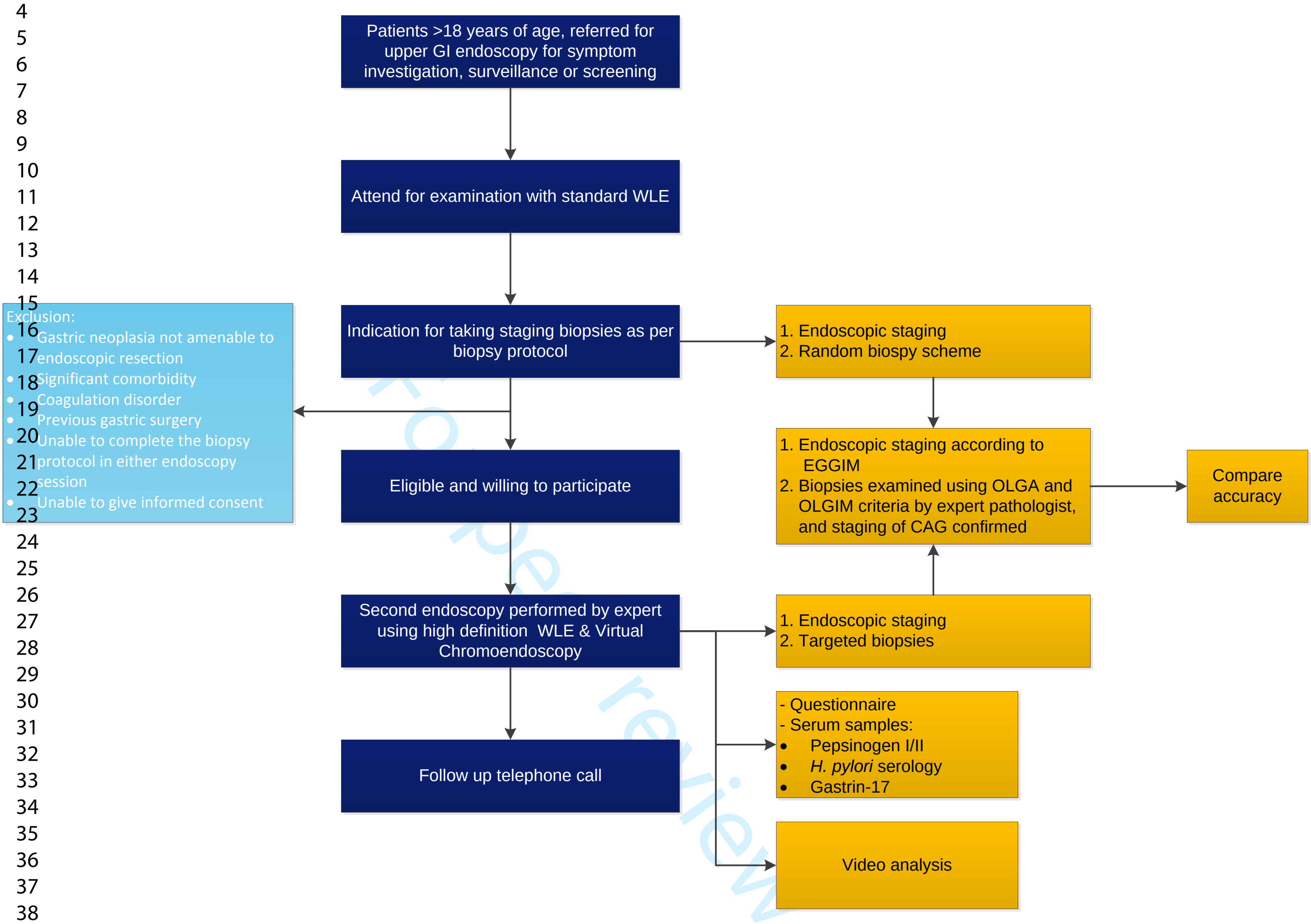

Figure 1 Flowchart of the study design; *CAG* chronic atrophic gastritis, *EGGIM* endoscopic grading of gastric intestinal metaplasia *GI* gastrointestinal, *H. pylori* Helicobacter pylori, *OLGA* operative link for gastritis assessment, *OLGIM* operative link for gastric intestinal metaplasia assessment, *WLE* white light endoscopy

For peer review only

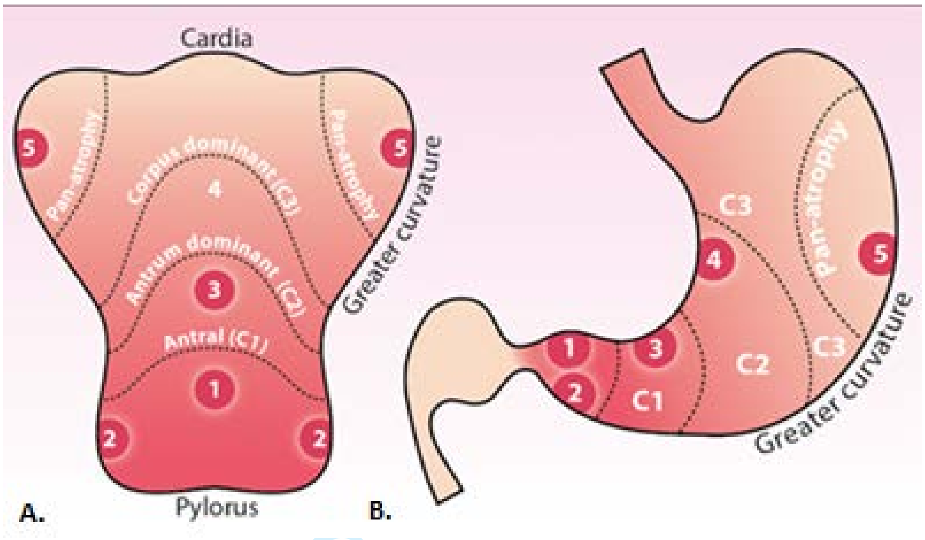

Figure 2 Biopsy strategy. *A.) Sydney protocol biopsy sites in the opened stomach along the greater curvature; B.) Biopsy sites in the anatomical view.*

For peer review only

Section & Topic	No	Item	Reported on page #
**TITLE OR ABSTRACT**			
	**1**	Identification as a study of diagnostic accuracy using at least one measure of accuracy (such as sensitivity, specificity, predictive values, or AUC)	1
**ABSTRACT**			
	**2**	Structured summary of study design, methods, results, and conclusions (for specific guidance, see STARD for Abstracts)	2
**INTRODUCTION**			
	**3**	Scientific and clinical background, including the intended use and clinical role of the index test	4
	**4**	Study objectives and hypotheses	6
**METHODS**			
Study design	**5**	Whether data collection was planned before the index test and reference standard were performed (prospective study) or after (retrospective study)	6
Participants	**6**	Eligibility criteria	7
	**7**	On what basis potentially eligible participants were identified (such as symptoms, results from previous tests, inclusion in registry)	7
	**8**	Where and when potentially eligible participants were identified (setting, location and dates)	7
	**9**	Whether participants formed a consecutive, random or convenience series	7
Test methods	**10a**	Index test, in sufficient detail to allow replication	8
	**10b**	Reference standard, in sufficient detail to allow replication	8
	**11**	Rationale for choosing the reference standard (if alternatives exist)	4
	**12a**	Definition of and rationale for test positivity cut-offs or result categories of the index test, distinguishing pre-specified from exploratory	9
	**12b**	Definition of and rationale for test positivity cut-offs or result categories of the reference standard, distinguishing pre-specified from exploratory	9
	**13a**	Whether clinical information and reference standard results were available to the performers/readers of the index test	4
	**13b**	Whether clinical information and index test results were available to the assessors of the reference standard	4
Analysis	**14**	Methods for estimating or comparing measures of diagnostic accuracy	7-9
	**15**	How indeterminate index test or reference standard results were handled	9
	**16**	How missing data on the index test and reference standard were handled	9
	**17**	Any analyses of variability in diagnostic accuracy, distinguishing pre-specified from exploratory	9
	**18**	Intended sample size and how it was determined	9
**RESULTS**			
Participants	**19**	Flow of participants, using a diagram	Fig 1
	**20**	Baseline demographic and clinical characteristics of participants	Protocol, i.e. no results provided yet
	**21a**	Distribution of severity of disease in those with the target condition	Protocol, i.e. no results provided yet
	**21b**	Distribution of alternative diagnoses in those without the target condition	Protocol, i.e. no results provided yet
	**22**	Time interval and any clinical interventions between index test and reference standard	Protocol, i.e. no results provided yet
Test results	**23**	Cross tabulation of the index test results (or their distribution) by the results of the reference standard	Protocol, i.e. no results provided yet
	**24**	Estimates of diagnostic accuracy and their precision (such as 95% confidence intervals)	Protocol, i.e. no results provided yet
	**25**	Any adverse events from performing the index test or the reference standard	Protocol, i.e. no results provided yet
**DISCUSSION**			
	**26**	Study limitations, including sources of potential bias, statistical uncertainty, and generalisability	11
	**27**	Implications for practice, including the intended use and clinical role of the index test	11
**OTHER INFORMATION**			

For peer review only - http://bmjopen.bmj.com/site/about/guidelines.xhtml

CC BY SA

	**28**	Registration number and name of registry	2
	**29**	Where the full study protocol can be accessed	2
	**30**	Sources of funding and other support; role of funders	11

For peer review only

STARD 2015

AIM

STARD stands for "Standards for Reporting Diagnostic accuracy studies". This list of items was developed to contribute to the completeness and transparency of reporting of diagnostic accuracy studies. Authors can use the list to write informative study reports. Editors and peer-reviewers can use it to evaluate whether the information has been included in manuscripts submitted for publication.

EXPLANATION

A **diagnostic accuracy study** evaluates the ability of one or more medical tests to correctly classify study participants as having a **target condition.** This can be a disease, a disease stage, response or benefit from therapy, or an event or condition in the future. A medical test can be an imaging procedure, a laboratory test, elements from history and physical examination, a combination of these, or any other method for collecting information about the current health status of a patient.

The test whose accuracy is evaluated is called **index test.** A study can evaluate the accuracy of one or more index tests. Evaluating the ability of a medical test to correctly classify patients is typically done by comparing the distribution of the index test results with those of the **reference standard**. The reference standard is the best available method for establishing the presence or absence of the target condition. An accuracy study can rely on one or more reference standards.

If test results are categorized as either positive or negative, the cross tabulation of the index test results against those of the reference standard can be used to estimate the **sensitivity** of the index test (the proportion of participants *with* the target condition who have a positive index test), and its **specificity** (the proportion *without* the target condition who have a negative index test). From this cross tabulation (sometimes referred to as the contingency or "2x2" table), several other accuracy statistics can be estimated, such as the positive and negative **predictive values** of the test. Confidence intervals around estimates of accuracy can then be calculated to quantify the statistical **precision** of the measurements.

If the index test results can take more than two values, categorization of test results as positive or negative requires a **test positivity cut-off**. When multiple such cut-offs can be defined, authors can report a receiver operating characteristic (ROC) curve which graphically represents the combination of sensitivity and specificity for each possible test positivity cut-off. The **area under the ROC curve** informs in a single numerical value about the overall diagnostic accuracy of the index test.

The **intended use** of a medical test can be diagnosis, screening, staging, monitoring, surveillance, prediction or prognosis. The **clinical role** of a test explains its position relative to existing tests in the clinical pathway. A replacement test, for example, replaces an existing test. A triage test is used before an existing test; an add-on test is used after an existing test.

Besides diagnostic accuracy, several other outcomes and statistics may be relevant in the evaluation of medical tests. Medical tests can also be used to classify patients for purposes other than diagnosis, such as staging or prognosis. The STARD list was not explicitly developed for these other outcomes, statistics, and study types, although most STARD items would still apply.

DEVELOPMENT

This STARD list was released in 2015. The 30 items were identified by an international expert group of methodologists, researchers, and editors. The guiding principle in the development of STARD was to select items that, when reported, would help readers to judge the potential for bias in the study, to appraise the applicability of the study findings and the validity of conclusions and recommendations. The list represents an update of the first version, which was published in 2003.

More information can be found on http://www.equator-network.org/reporting-guidelines/stard.

For peer review only - http://bmjopen.bmj.com/site/about/guidelines.xhtml

BMJ Open

ACCURACY OF ENDOSCOPIC STAGING AND TARGETED BIOPSIES FOR ROUTINE GASTRIC INTESTINAL METAPLASIA AND GASTRIC ATROPHY EVALUATION: STUDY PROTOCOL OF A PROSPECTIVE, COHORT STUDY – THE ESTIMATE STUDY -

Journal:	*BMJ Open*
Manuscript ID	bmjopen-2019-032013.R2
Article Type:	Protocol
Date Submitted by the Author:	26-Aug-2019
Complete List of Authors:	Nieuwenburg, Stella; Erasmus MC University Medical Center, Gastroenterology & Hepatology<br>Waddingham, William; University College London Hospital, Endoscopy; UCL Cancer Institute<br>Graham, David; University College London Hospital, Endoscopy<br>Rodriguez-Justo, Manuel; University College London Hospital, Pathology<br>Biermann, Katharina; Erasmus MC University Medical Center, Pathology<br>Kuipers, Ernst; Erasmus MC University Medical Center, Gastroenterology & Hepatology<br>Banks, Matthew; University College London Hospital, Endoscopy<br>Jansen, Marnix; University College London Hospital, Endoscopy; University College London Hospital, Pathology<br>Spaander, Manon; Erasmus MC University Medical Center, Gastroenterology &Hepatology
**Primary Subject Heading**:	Gastroenterology and hepatology
Secondary Subject Heading:	Oncology
Keywords:	Endoscopy < GASTROENTEROLOGY, Gastrointestinal tumours < GASTROENTEROLOGY, PREVENTIVE MEDICINE

SCHOLARONE™ Manuscripts

ACCURACY OF ENDOSCOPIC STAGING AND TARGETED BIOPSIES FOR ROUTINE GASTRIC INTESTINAL METAPLASIA AND GASTRIC ATROPHY EVALUATION STUDY PROTOCOL OF A PROSPECTIVE, COHORT STUDY

# – THE ESTIMATE STUDY -

Stella A.V. Nieuwenburg[1]*, William W. Waddingham[2,3]*, David Graham[2], Manuel Rodriguez-Justo[3,4], Katharina Biermann[5], Ernst J. Kuipers[1], Matthew R. Banks[2], Marnix Jansen[2,4], Manon C.W. Spaander[1]

** Authors share first authorship*

[1] *Department of Gastroenterology and Hepatology, Erasmus MC University Medical Center, Rotterdam, the Netherlands*

[2] *Department of Endoscopy, University College London Hospital, London, UK*

[3] *UCL Cancer Institute, University College London, London, UK*

[4] *Department of Pathology, University College London, London, UK*

[5] *Department of Pathology, Erasmus MC University Medical Center, Rotterdam, The Netherlands*

CORRESPONDENCE:

Manon C.W. Spaander, MD, PhD, Associate professor

Department of Gastroenterology and Hepatology (room Hs-308)

Erasmus MC University Medical Center

‘s Gravendijkwal 230, 3015 CE Rotterdam, The Netherlands

Telephone: +31 (0) 10 7035643 Fax: +31 (0) 10 7035172

E-mail: v.spaander@erasmusmc.nl

For peer review only

Abstract

Introduction Patients with chronic atrophic gastritis (CAG) and intestinal metaplasia (IM) are at risk of developing gastric adenocarcinoma. Their diagnosis and management currently relies on histopathological guidance after random endoscopic biopsy sampling (Sydney biopsy strategy). This approach has significant flaws such as under-diagnosis, poor reproducibility and poor correlation between endoscopy and histology. This prospective, international multicentre study aims to establish whether endoscopy-led risk stratification accurately and reproducibly predicts CAG and IM extent and disease stage.

Methods and analysis Patients with CAG and/or IM on standard white light endoscopy (WLE) will be prospectively identified and invited to undergo a second endoscopy performed by an expert endoscopist using enhanced endoscopic imaging techniques with virtual chromoendoscopy. Extent of CAG/IM will be endoscopically staged with enhanced imaging and compared to standard WLE. Histopathologic risk stratification through targeted biopsies will be compared to endoscopic disease staging and to random biopsy staging on WLE as a reference. At least 234 patients are required to show a 10% difference in sensitivity and accuracy between enhanced imaging endoscopy-led staging and the current biopsy-led staging protocol of gastric atrophy with a power (beta) of 80% and a 0.05 probability of a type I error (alpha).

Ethics and dissemination The study was approved by the respective Institutional Review Boards (Netherlands: MEC-2018-078; UK: 19/LO/0089). The findings will be published in peer-reviewed journals and presented at scientific meetings.

Trial registration number NTC=7661

For peer review only

Strengths and limitations of this study

- This is the first study to compare endoscopy-led risk stratification of premalignant gastric lesions using advanced imaging and targeted biopsies with white light endoscopy and random biopsies as a reference, performed at separate occasions with the endoscopist blinded to prior results.
- This study will additionally provide biobank biopsy and serum material for future biomarker analysis.
- A possible limitation of this study is that all procedures will be performed by expert endoscopists in teaching hospitals, therefore external reproducibility will be evaluated using interobserver variability of disease staging through video recordings.
- The same limitation holds for the histopathological evaluation of the biopsy samples for which a proportion of the samples will be reviewed and re-scored by a blinded second expert GI histopathologist.

For peer review only

Introduction

Gastric adenocarcinoma remains a major cause of cancer mortality and is the most commonly diagnosed malignant condition of the gastrointestinal tract (1-4). Although incidence rates had previously been declining, recent studies demonstrated this differs among population subgroups. For example, an increasing incidence of gastric adenocarcinoma among young white cohorts in Western countries was objectified. This may be due to an increasing prevalence of gastric cancer precursors among younger adults, in particular chronic atrophic gastritis (CAG),intestinal metaplasia (IM), and dysplasia (5, 6). These studies suggest that gastric cancer incidence rates may plateau or even increase again in the upcoming years. Importantly, with the exception of Japan and Korea, the majority of gastric cancers worldwide are diagnosed at a late stage. This results in a poor prognosis with a less than 30% 5-year survival (1, 2, 7). Japan's earlier stage of diagnosis and superior 5-year survival highlight the need for earlier recognition and treatment (8).

Endoscopic recognition of the premalignant stomach has long been problematic and limited by the ability of endoscopist and the imaging tools. A previous study demonstrated that 22% of high-grade dysplastic (HGD) lesions and early gastric cancers were missed (9, 10). A meta-analysis and systematic review of endoscopy follow-up studies confirmed that a marked proportion of early gastric cancers are missed at endoscopy (10). Therefore, current practice uses histology based staging (11, 12). However, endoscopic imaging has significantly improved with high-definition endoscopes and imaging enhancement technologies now routinely available. Some recent studies already suggested that accurate endoscopic staging of CAG and GIM is achievable and can robustly predict gastric adenocarcinoma risk. Importantly, the interobserver and intraobserver reproducibility characteristics of endoscopic CAG and GIM severity assessment are in experienced hands moderate to excellent (13-18). These marked improvements in endoscopic technology and the shift towards an endoscopy-led approach will empower the endoscopist to risk stratify individuals with greater accuracy and decrease the already huge burden placed on our

For peer review only

endoscopy and histopathology departments. Therefore, the aim of this study is to evaluate if enhanced endoscopic imaging, including high-definition white light endoscopy and virtual chromoendoscopy, alongside targeted biopsies, provides an accurate and reproducible assessment of CAG and IM disease extent and staging, when compared to the current practice of white light endoscopy and random biopsies through the Sydney protocol biopsy strategy.

For peer review only

Methods and analysis

AIMS

The primary aim of this study is to assess the diagnostic accuracy for the endoscopic diagnosis of intestinal metaplasia in Sydney biopsy locations comparing standard endoscopic staging with random biopsies to enhanced imaging with biopsies targeted to GIM (19). The Standards for Reporting of Diagnostic Accuracy Studies (STARD) guidelines were followed(20). Study sites are located in the Netherlands and the United Kingdom. Secondary objectives are to evaluate a) reproducibility of endoscopic staging after expert review, b) reproducibility of histopathology for detection of intestinal metaplasia, c) the number of dysplastic or neoplastic lesions detected, and d) effects of inspection time of gastric mucosa on diagnostic accuracy.

DESIGN

This is a prospective, multicentre registry study on the accuracy and reproducibility of enhanced endoscopic imaging, including high-definition white light endoscopy and virtual chromoendoscopy, for the staging of chronic atrophic gastritis and intestinal metaplasia. Two upper endoscopies will be performed on two separate occasions (6-12 months in between) using standard white-light endoscopy plus random biopsies (current diagnostic strategy) at the first endoscopy and enhanced endoscopic imaging with targeted biopsies (proposed diagnostic strategy) at the second endoscopy. We will compare both approaches using histopathology as a reference and assess the accuracy and reproducibility of enhanced endoscopic imaging (**Fig 1**). The study protocol was approved by respective Institutional Review Boards (Netherlands: MEC-2018-078; UK: 19/LO/0089). The protocol has been registered in the Dutch trial register (NTR=7661).

For peer review only

PATIENT AND PUBLIC INVOLVEMENT

We maintained close links with patient alliances and interest groups, both in the Netherlands as well as in the UK. This close relationship informs our practice and is the basis for the current study design. We will engage closely with patient interest groups to communicate research findings and ensure that our deliverables are fit for purpose.

PARTICIPANTS

Sample size

For estimation of sample size we assume that the diagnosis of CAG or IM on enhanced imaging and targeted biopsies must be set with at least a 90% sensitivity with white light endoscopy and random biopsies as a reference (15). A power (beta) of 80% and a probability of type I error (alpha) of 0.05 will be handled. That purpose requires at least 234 patients to be recruited to show a 10% difference in sensitivity between enhanced endoscopy-led staging and the standard WLE.

Recruitment

All patients (> 18 years of age) referred to the endoscopy department for routine diagnostic upper gastrointestinal endoscopy and diagnosed with chronic atrophic gastritis or intestinal metaplasia between November 2018 and June 2020 are eligible for inclusion if able to give informed consent. Patients are excluded when having 1) gastric neoplasia not amenable to endoscopic resection, 2) no indication for Sydney biopsy staging on standard WLE, 3) significant comorbidity, 4) a coagulation disorder, 5) previous gastric surgery, or 6) are unable to complete the biopsy protocol in either endoscopy session.

INTERVENTIONS

Baseline characteristics

All patients are asked to complete a questionnaire on lifestyle factors, medical history, past interventions, medication use and family history of gastric cancer.

For peer review only - http://bmjopen.bmj.com/site/about/guidelines.xhtml

White light endoscopy

Patients referred to the endoscopy department for upper gastrointestinal endoscopy for investigation of symptoms or for surveillance of a known condition will undergo their procedure on a standard diagnostic gastroscopy list. Patients found to have chronic atrophic gastritis or intestinal metaplasia will be prospectively identified. During the initial procedure patients will receive the current recommended practice. Current practice is to initially identify if gastric atrophy is present and to inspect the gastric mucosa for areas suspicious for dysplasia or malignancy. Any mucosal abnormalities suspicious for dysplasia or malignancy are biopsied with tissue biopsies placed in separate containers. Following this, ten random biopsies are taken according to the Sydney protocol (see also **Fig. 2**): 4 quadrant biopsies of the antrum, 2 biopsies from the incisura, and 4 biopsies from the body of the stomach, respectively two from the lesser curve, and two from the greater curve.

Enhanced imaging endoscopy

Patients who opt to be recruited to the study will be invited for a second endoscopy at 6 to 12-months interval. This will be performed by one of the experts on this protocol. The endoscopists will be blinded to any previous endoscopy or biopsy results. This second endoscopy will be recorded and performed using enhanced endoscopic imaging. Given that these patients will have recently undergone a complete upper gastrointestinal endoscopy, whilst all anatomical landmarks will be viewed, the focus of this examination will be on the gastric mucosa. The endoscopist will record 1) the extent of gastric atrophy, 2) the presence and extent of intestinal metaplasia in each of the aforementioned areas. This will be done using our simplified endoscopic metaplasia scoring system (GRAHAM score) (**Table 1**). Biopsies will then be taken in the following manner: 1) Areas of intestinal metaplasia found in any of the Sydney protocol areas, 2) Sydney areas negative for gastric intestinal metaplasia will be randomly biopsied, as control, to complete the assessment, 3) Lesions suspicious for dysplasia or malignancy.

For peer review only

	Focal / Minimal metaplasia (<1/3 of surface coverage)	Moderate / extensive metaplasia (>1/3 surface coverage)
**Antrum & incisura**	1	2
**Lesser curve**	1	2
**Greater curve**	1	2

Table 1 Simplified endoscopic gastric intestinal metaplasia staging system: “GRAHAM Score”

Histopathological assessment

Each biopsy will be reviewed at the teaching hospital by one of the expert gastrointestinal histopathologists named on this protocol according to the established OLGA and OLGIM staging systems (21). Histopathologists will be blinded to whether biopsies were directed at areas suspicious for intestinal metaplasia and to the biopsy results of WLE staging. A proportion of biopsy samples will be reviewed and re-scored by a second expert GI histopathologist, who is blinded to the initial results. This is to ensure inter-observer reproducibility for histopathological detection of intestinal metaplasia.

Serology assessment

A proportion of the collected serum will be used to assess *Helicobacter pylori (H. pylori)* serology, pepsinogen I/II ratio and gastrin-17. The remaining serum will then be stored for use in future studies exploring the development of molecular biomarkers for gastric atrophy risk stratification.

Data collection and management

All data collected for this study will be recorded in an anonymised format on a centralised, secure web-based platform (OpenClinica). Source data will be recorded in patients’ notes or electronic health records, and hard copies of consent forms will be stored in a secure locked cabinet per site. All study data will be stored in a linked anonymised fashion against a study number, with the registry of study numbers stored separately on an encrypted database.

For peer review only

Statistical analyses

For descriptive statistics, mean (±SD) will be used in case of a normal distribution of variables and median (25%-75%) will be used for variables with a skewed distribution. Where appropriate, the Student t test or Mann-Whitney U test will be used.

Diagnostic accuracy of endoscopic diagnosis of chronic atrophic gastritis and intestinal metaplasia is defined as the total number of directed biopsies that confirm the endoscopic impression of the presence or absence of intestinal metaplasia divided by the total number of biopsies (accuracy = true positives + true negatives / all biopsies). Results will be compared to the histopathology outcomes using the chi-squared test after multiple testing correction as well as kappa values for interobserver agreement among endoscopists and histopathologists.

After study completion, all videos will be collated and anonymised prior to expert panel review and estimation of the severity and extent of atrophy as well as intestinal metaplasia. 5 expert reviewers will review 50 videos each (Kappa 0.4) for the purposes of assessing inter-observer reliability. Sensitivity, specificity, and global accuracy along with the 95% confidence intervals will be established. Duration of inspection time of gastric mucosa and its relation to diagnostic accuracy will be evaluated using, when appropriate, a paired t-test or Wilcoxon test. All tests will be two-sided.

Ethics and dissemination

The study was approved by the Institutional Review Boards of the Erasmus Medical Center (Erasmus MC) and University College London Hospitals (UCLH) (Netherlands: MEC-2018-078; UK: 19/LO/0089).

Results will be disseminated to potential users in academia and medical industries, through the standard routes of presentations, oral and posters, at local, national and international conferences, undergraduate and graduate teaching and through peer-reviewed publication. Efforts will be made to present work in a timely manner at key international meetings to encourage collaboration with research partners.

For peer review only

Discussion

The recently updated European MAPS guidelines recommend surveillance of patients with premalignant gastric mucosal lesions by performing endoscopy (preferably with advanced imaging) and taking random biopsies of the stomach for histopathological assessment. This enables the detection of progression to high-risk lesions and eventually cancer (22). However, various studies indicate that a marked proportion of advanced gastric lesions are missed at a stage when these lesions are potentially still amenable to endoscopic management. This implies that the risk of undertreatment is undeniable (9). The development of high-definition endoscopy and virtual chromoendoscopy has been a main focus of research in the past years and it has revolutionised the endoscopic assessment of the premalignant stomach by being superior to white light imaging. (23). The updated MAPS guidelines opts for the use of advanced imaging as the preferred surveillance method. Recently, Esposito G et al showed a scoring tool based on endoscopic staging using Endoscopic Grading of Gastric Intestinal Metaplasia (EGGIM) with advanced imaging as a promising decision tool to identify patients at risk of gastric cancer (24). However, currently there are no studies on how the use of advanced endoscopic imaging to detect IM of the stomach can be applied in countries with a low prevalence of IM. Still, histological confirmation is needed through random biopsies. Future steps are to evaluate the possible shift towards an endoscopy-led strategy now these marked improvements in endoscopic technology are within our reach. This prospective study was therefore designed to determine the validity of endoscopy-led staging of the premalignant stomach using advanced imaging and taking targeted biopsies for histological confirmation.

A previous comparative study between white light and high-definition endoscopy for the diagnosis of premalignant gastric lesions indeed showed a superior diagnostic accuracy of high-definition endoscopy (15). However, one limitation was that WLE and high-definition endoscopy were performed during one occasion, which implied that the endoscopist was not blinded to the WLE results. Within the current protocol, we choose to perform the procedures on two separate occasions with blinding of the expert endoscopist to the previous WLE

For peer review only

results.

Over the years serological markers have shown major promise for predicting the presence and severity of gastric premalignant lesions (25-27). Pepsinogens are serological markers for atrophy in the stomach and can be divided into pepsinogen I and II. A decreased PG I/II ratio indicates the presence of atrophic changes. Gastrin serum levels are indicative for gastric acd output and are increased in the presence of atrophic changes (27). The collection of serum samples was included in our protocol to strengthen risk stratification for progression of premalignant gastric lesions.

A few limitations of the study should be mentioned. All high-definition endoscopies will be performed by expert endoscopists at either site. A potential caveat with this design is the generalisability of the study outcomes to non-expert settings. To test this, we selected a panel of independent endoscopists who all will review recorded endoscopy videos in order to assess inter-observer variability. The same limitation holds for the histopathological evaluation of the biopsy samples. Therefore, a proportion of the samples will be reviewed and re-scored by a blinded second expert GI histopathologist.

In conclusion, prospective validation of endoscopy-led staging of the premalignant stomach will provide the needed evidence for an endoscopy-led risk stratification of patients at risk for gastric adenocarcinoma. This will allow rational design of tiered screening and surveillance protocols to benefit early-stage gastric cancer detection within at-risk populations. This will major implications for affected patients and general health care resource utilisation.

Funding statement

This work was supported by a grant from the Maag Lever Darm Stichting, Dutch Digestive Foundation grant number D17-22.

Competing interest statement

None declared.

For peer review only

Contributorship

EJK and MRB conceived the idea for the study, designed the protocol and supervise study execution. MCWS, DG and MJ supervise study execution. SAVN and WW drafted the manuscript. EJK, MRB, DG, MJ, MCWS, SAVN and WW analyse and interpret data. MRJ and KB interpret data. All authors provided critical revision of the manuscript for important intellectual content and approved the final draft of the protocol for submission.

For peer review only

References

1. Stomach cancer incidence statistics 2015 [Available from: http://www.cancerresearchuk.org/cancer-info/cancerstats.

2. Global cancer statistics: World Health Organization; [Available from: https://www.who.int/cancer/en/.

3. Amiri M, Janssen F, Kunst AE. The decline in stomach cancer mortality: exploration of future trends in seven European countries. Eur J Epidemiol. 2011;26(1):23-8.

4. Rahman R, Asombang AW, Ibdah JA. Characteristics of gastric cancer in Asia. World J Gastroenterol. 2014;20(16):4483-90.

5. Song H, Held M, Sandin S, Rautelin H, Eliasson M, Soderberg S, et al. Increase in the Prevalence of Atrophic Gastritis Among Adults Age 35 to 44 Years Old in Northern Sweden Between 1990 and 2009. Clin Gastroenterol Hepatol. 2015;13(9):1592-600 e1.

6. Anderson WF, Camargo MC, Fraumeni JF, Jr., Correa P, Rosenberg PS, Rabkin CS. Age-specific trends in incidence of noncardia gastric cancer in US adults. Jama. 2010;303(17):1723-8.

7. Maconi G, Manes G, Porro GB. Role of symptoms in diagnosis and outcome of gastric cancer. World J Gastroenterol. 2008;14(8):1149-55.

8. Matsuda T, Saika K. The 5-year relative survival rate of stomach cancer in the USA, Europe and Japan. Jpn J Clin Oncol. 2013;43(11):1157-8.

9. Ren W, Yu J, Zhang ZM, Song YK, Li YH, Wang L. Missed diagnosis of early gastric cancer or high-grade intraepithelial neoplasia. World J Gastroenterol. 2013;19(13):2092-6.

10. Menon S, Trudgill N. How commonly is upper gastrointestinal cancer missed at endoscopy? A meta-analysis. Endosc Int Open. 2014;2(2):E46-50.

11. Dinis-Ribeiro M, Areia M, de Vries AC, Marcos-Pinto R, Monteiro-Soares M, O'Connor A, et al. Management of precancerous conditions and lesions in the stomach (MAPS): guideline from the European Society of Gastrointestinal Endoscopy (ESGE), European Helicobacter Study Group (EHSG), European Society of Pathology (ESP), and the Sociedade Portuguesa de Endoscopia Digestiva (SPED). Endoscopy. 2012;44(1):74-94.

12. Yue H, Shan L, Bin L. The significance of OLGA and OLGIM staging systems in the risk assessment of gastric cancer: a systematic review and meta-analysis. Gastric Cancer. 2018;21(4):579-87.

13. Miwata T, Quach DT, Hiyama T, Aoki R, Le HM, Tran PL, et al. Interobserver and intraobserver agreement for gastric mucosa atrophy. BMC Gastroenterol. 2015;15:95.

14. Toyoshima O, Yamaji Y, Yoshida S, Matsumoto S, Yamashita H, Kanazawa T, et al. Endoscopic gastric atrophy is strongly associated with gastric cancer development after Helicobacter pylori eradication. Surg Endosc. 2017;31(5):2140-8.

For peer review only

15. Pimentel-Nunes P, Libanio D, Lage J, Abrantes D, Coimbra M, Esposito G, et al. A multicenter prospective study of the real-time use of narrow-band imaging in the diagnosis of premalignant gastric conditions and lesions. Endoscopy. 2016;48(8):723-30.

16. Xirouchakis E, Laoudi F, Tsartsali L, Spiliadi C, Georgopoulos SD. Screening for gastric premalignant lesions with narrow band imaging, white light and updated Sydney protocol or both? Dig Dis Sci. 2013;58(4):1084-90.

17. Uedo N, Ishihara R, Iishi H, Yamamoto S, Yamamoto S, Yamada T, et al. A new method of diagnosing gastric intestinal metaplasia: narrow-band imaging with magnifying endoscopy. Endoscopy. 2006;38(8):819-24.

18. Banks M, Graham D, Jansen M, Gotoda T, Coda S, di Pietro M, et al. British Society of Gastroenterology guidelines on the diagnosis and management of patients at risk of gastric adenocarcinoma. Gut. 2019.

19. Dixon MF, Genta RM, Yardley JH, Correa P. Classification and grading of gastritis. The updated Sydney System. International Workshop on the Histopathology of Gastritis, Houston 1994. Am J Surg Pathol. 1996;20(10):1161-81.

20. Cohen JF, Korevaar DA, Altman DG, Bruns DE, Gatsonis CA, Hooft L, et al. STARD 2015 guidelines for reporting diagnostic accuracy studies: explanation and elaboration. BMJ Open. 2016;6(11):e012799.

21. Cho SJ, Choi IJ, Kook MC, Nam BH, Kim CG, Lee JY, et al. Staging of intestinal- and diffuse-type gastric cancers with the OLGA and OLGIM staging systems. Aliment Pharmacol Ther. 2013;38(10):1292-302.

22. Pimentel-Nunes P, Libanio D, Marcos-Pinto R, Areia M, Leja M, Esposito G, et al. Management of epithelial precancerous conditions and lesions in the stomach (MAPS II): European Society of Gastrointestinal Endoscopy (ESGE), European Helicobacter and Microbiota Study Group (EHMSG), European Society of Pathology (ESP), and Sociedade Portuguesa de Endoscopia Digestiva (SPED) guideline update 2019. Endoscopy. 2019.

23. East JE, Vleugels JL, Roelandt P, Bhandari P, Bisschops R, Dekker E, et al. Advanced endoscopic imaging: European Society of Gastrointestinal Endoscopy (ESGE) Technology Review. Endoscopy. 2016;48(11):1029-45.

24. Esposito G, Pimentel-Nunes P, Angeletti S, Castro R, Libanio D, Galli G, et al. Endoscopic grading of gastric intestinal metaplasia (EGGIM): a multicenter validation study. Endoscopy. 2019;51(6):515-21.

25. den Hollander WJ, Holster IL, den Hoed CM, Capelle LG, Tang TJ, Anten MP, et al. Surveillance of premalignant gastric lesions: a multicentre prospective cohort study from low incidence regions. Gut. 2018.

For peer review only - http://bmjopen.bmj.com/site/about/guidelines.xhtml

26. Huang YK, Yu JC, Kang WM, Ma ZQ, Ye X, Tian SB, et al. Significance of Serum Pepsinogens as a Biomarker for Gastric Cancer and Atrophic Gastritis Screening: A Systematic Review and Meta-Analysis. PLoS One. 2015;10(11):e0142080.

27. Leja M, Kupcinskas L, Funka K, Sudraba A, Jonaitis L, Ivanauskas A, et al. Value of gastrin-17 in detecting antral atrophy. Adv Med Sci. 2011;56(2):145-50.

For peer review only

Figure 1 Flowchart of the study design; *CAG* chronic atrophic gastritis, *EGGIM* endoscopic grading of gastric intestinal metaplasia *GI* gastrointestinal, *H. pylori* Helicobacter pylori, *OLGA* operative link for gastritis assessment, *OLGIM* operative link for gastric intestinal metaplasia assessment, *WLE* white light endoscopy

Figure 2 Biopsy strategy. *A.) Sydney protocol biopsy sites in the opened stomach along the greater curvature; B.) Biopsy sites in the anatomical view.*

For peer review only

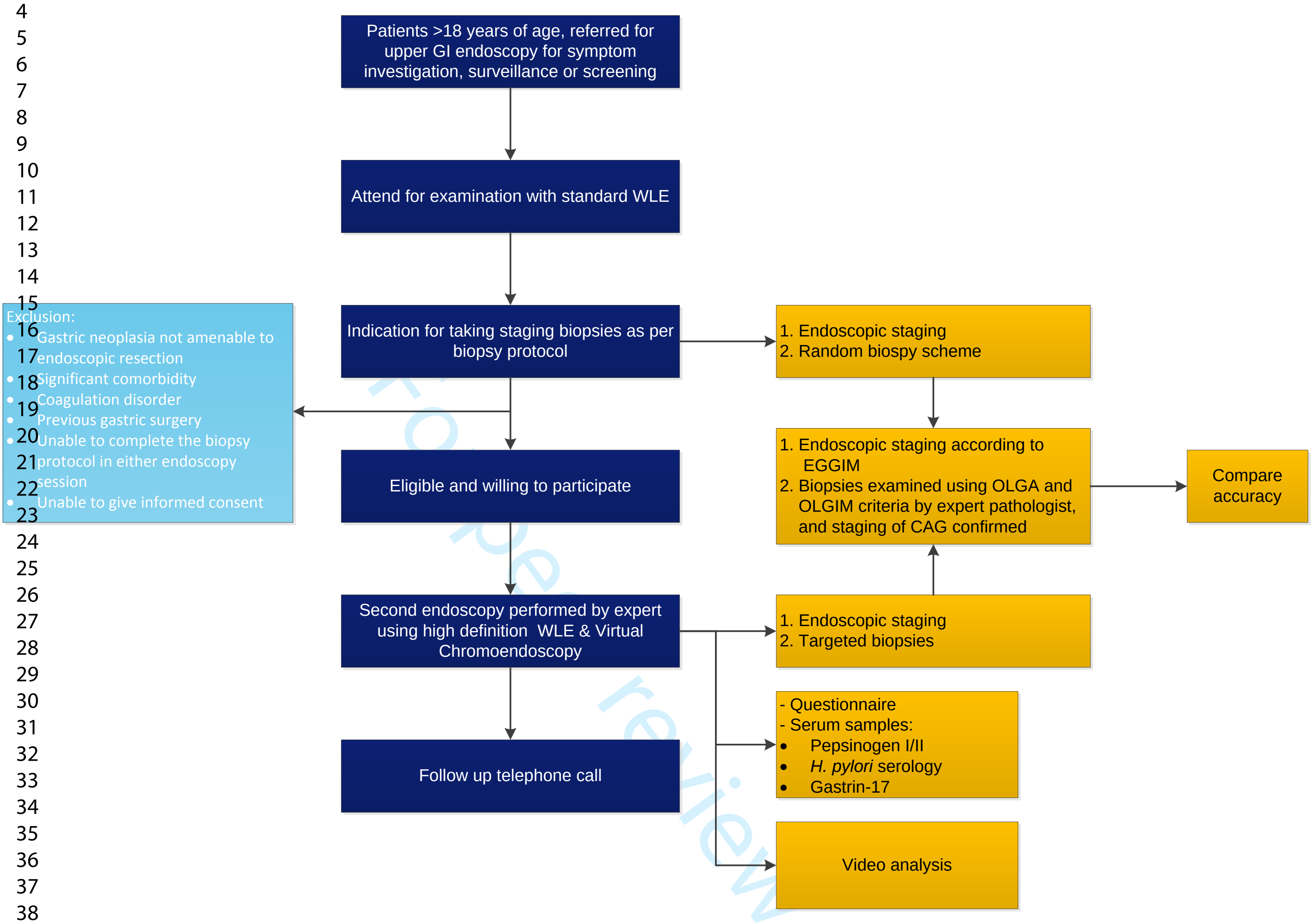

Figure 1 Flowchart of the study design; *CAG* chronic atrophic gastritis, *EGGIM* endoscopic grading of gastric intestinal metaplasia *GI* gastrointestinal, *H. pylori* Helicobacter pylori, *OLGA* operative link for gastritis assessment, *OLGIM* operative link for gastric intestinal metaplasia assessment, *WLE* white light endoscopy

For peer review only

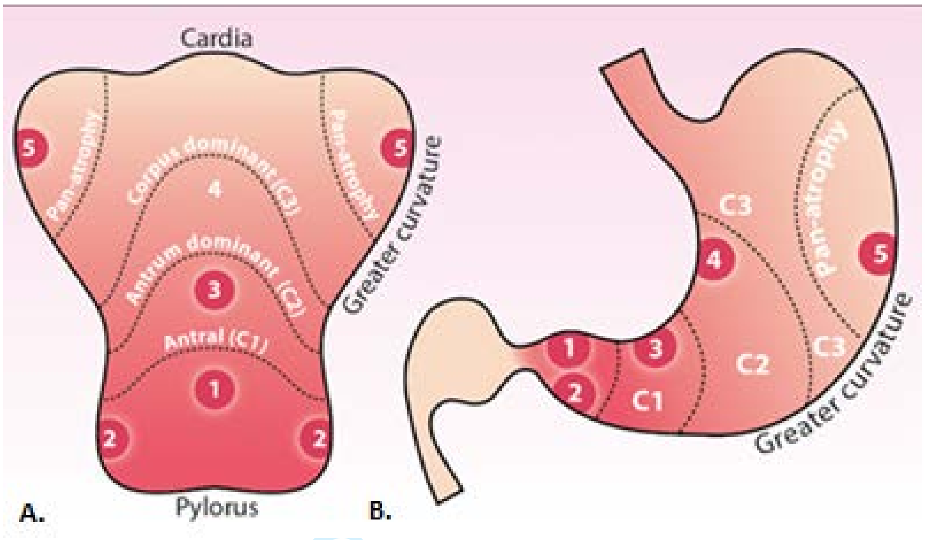

Figure 2 Biopsy strategy. *A.) Sydney protocol biopsy sites in the opened stomach along the greater curvature; B.) Biopsy sites in the anatomical view.*

Section & Topic	No	Item	Reported on page #
**TITLE OR ABSTRACT**			
	**1**	Identification as a study of diagnostic accuracy using at least one measure of accuracy (such as sensitivity, specificity, predictive values, or AUC)	1
**ABSTRACT**			
	**2**	Structured summary of study design, methods, results, and conclusions (for specific guidance, see STARD for Abstracts)	2
**INTRODUCTION**			
	**3**	Scientific and clinical background, including the intended use and clinical role of the index test	4
	**4**	Study objectives and hypotheses	6
**METHODS**			
Study design	**5**	Whether data collection was planned before the index test and reference standard were performed (prospective study) or after (retrospective study)	6
Participants	**6**	Eligibility criteria	7
	**7**	On what basis potentially eligible participants were identified (such as symptoms, results from previous tests, inclusion in registry)	7
	**8**	Where and when potentially eligible participants were identified (setting, location and dates)	7
	**9**	Whether participants formed a consecutive, random or convenience series	7
Test methods	**10a**	Index test, in sufficient detail to allow replication	8
	**10b**	Reference standard, in sufficient detail to allow replication	8
	**11**	Rationale for choosing the reference standard (if alternatives exist)	4
	**12a**	Definition of and rationale for test positivity cut-offs or result categories of the index test, distinguishing pre-specified from exploratory	9
	**12b**	Definition of and rationale for test positivity cut-offs or result categories of the reference standard, distinguishing pre-specified from exploratory	9
	**13a**	Whether clinical information and reference standard results were available to the performers/readers of the index test	4
	**13b**	Whether clinical information and index test results were available to the assessors of the reference standard	4
Analysis	**14**	Methods for estimating or comparing measures of diagnostic accuracy	7-9
	**15**	How indeterminate index test or reference standard results were handled	9
	**16**	How missing data on the index test and reference standard were handled	9
	**17**	Any analyses of variability in diagnostic accuracy, distinguishing pre-specified from exploratory	9
	**18**	Intended sample size and how it was determined	9
**RESULTS**			
Participants	**19**	Flow of participants, using a diagram	Fig 1
	**20**	Baseline demographic and clinical characteristics of participants	Protocol, i.e. no results provided yet
	**21a**	Distribution of severity of disease in those with the target condition	Protocol, i.e. no results provided yet
	**21b**	Distribution of alternative diagnoses in those without the target condition	Protocol, i.e. no results provided yet
	**22**	Time interval and any clinical interventions between index test and reference standard	Protocol, i.e. no results provided yet
Test results	**23**	Cross tabulation of the index test results (or their distribution) by the results of the reference standard	Protocol, i.e. no results provided yet
	**24**	Estimates of diagnostic accuracy and their precision (such as 95% confidence intervals)	Protocol, i.e. no results provided yet
	**25**	Any adverse events from performing the index test or the reference standard	Protocol, i.e. no results provided yet
**DISCUSSION**			
	**26**	Study limitations, including sources of potential bias, statistical uncertainty, and generalisability	11
	**27**	Implications for practice, including the intended use and clinical role of the index test	11
**OTHER INFORMATION**			

For peer review only

	**28**	Registration number and name of registry	2
	**29**	Where the full study protocol can be accessed	2
	**30**	Sources of funding and other support; role of funders	11

For peer review only

STARD 2015

AIM

STARD stands for "Standards for Reporting Diagnostic accuracy studies". This list of items was developed to contribute to the completeness and transparency of reporting of diagnostic accuracy studies. Authors can use the list to write informative study reports. Editors and peer-reviewers can use it to evaluate whether the information has been included in manuscripts submitted for publication.

EXPLANATION

A **diagnostic accuracy study** evaluates the ability of one or more medical tests to correctly classify study participants as having a **target condition.** This can be a disease, a disease stage, response or benefit from therapy, or an event or condition in the future. A medical test can be an imaging procedure, a laboratory test, elements from history and physical examination, a combination of these, or any other method for collecting information about the current health status of a patient.

The test whose accuracy is evaluated is called **index test.** A study can evaluate the accuracy of one or more index tests. Evaluating the ability of a medical test to correctly classify patients is typically done by comparing the distribution of the index test results with those of the **reference standard**. The reference standard is the best available method for establishing the presence or absence of the target condition. An accuracy study can rely on one or more reference standards.

If test results are categorized as either positive or negative, the cross tabulation of the index test results against those of the reference standard can be used to estimate the **sensitivity** of the index test (the proportion of participants *with* the target condition who have a positive index test), and its **specificity** (the proportion *without* the target condition who have a negative index test). From this cross tabulation (sometimes referred to as the contingency or "2x2" table), several other accuracy statistics can be estimated, such as the positive and negative **predictive values** of the test. Confidence intervals around estimates of accuracy can then be calculated to quantify the statistical **precision** of the measurements.

If the index test results can take more than two values, categorization of test results as positive or negative requires a **test positivity cut-off**. When multiple such cut-offs can be defined, authors can report a receiver operating characteristic (ROC) curve which graphically represents the combination of sensitivity and specificity for each possible test positivity cut-off. The **area under the ROC curve** informs in a single numerical value about the overall diagnostic accuracy of the index test.

The **intended use** of a medical test can be diagnosis, screening, staging, monitoring, surveillance, prediction or prognosis. The **clinical role** of a test explains its position relative to existing tests in the clinical pathway. A replacement test, for example, replaces an existing test. A triage test is used before an existing test; an add-on test is used after an existing test.

Besides diagnostic accuracy, several other outcomes and statistics may be relevant in the evaluation of medical tests. Medical tests can also be used to classify patients for purposes other than diagnosis, such as staging or prognosis. The STARD list was not explicitly developed for these other outcomes, statistics, and study types, although most STARD items would still apply.

DEVELOPMENT

This STARD list was released in 2015. The 30 items were identified by an international expert group of methodologists, researchers, and editors. The guiding principle in the development of STARD was to select items that, when reported, would help readers to judge the potential for bias in the study, to appraise the applicability of the study findings and the validity of conclusions and recommendations. The list represents an update of the first version, which was published in 2003.

More information can be found on http://www.equator-network.org/reporting-guidelines/stard.

CC BY SA

For peer review only - http://bmjopen.bmj.com/site/about/guidelines.xhtml
